# Whole-Genome Sequencing of Chinese Yellow Catfish Provides a Valuable Genetic Resource for High-Throughput Identification of Toxin Genes

**DOI:** 10.3390/toxins10120488

**Published:** 2018-11-23

**Authors:** Shiyong Zhang, Jia Li, Qin Qin, Wei Liu, Chao Bian, Yunhai Yi, Minghua Wang, Liqiang Zhong, Xinxin You, Shengkai Tang, Yanshan Liu, Yu Huang, Ruobo Gu, Junmin Xu, Wenji Bian, Qiong Shi, Xiaohui Chen

**Affiliations:** 1 Freshwater Fisheries Research Institute of Jiangsu Province, Nanjing 210017, China; shiyongzhang@hotmail.com (S.Z.); qinqinapple1980@163.com (Q.Q.); wangminghua18@sina.com (M.W.); lqzhongffri@hotmail.com (L.Z.); tangshengkai1981@sohu.com (S.T.); liuyanshan613@sina.com (Y.L.); 2 BGI Education Center, University of Chinese Academy of Sciences, Shenzhen 518083, China; yiyunhai@genomics.cn (Y.Y.); huangyu@genomics.cn (Y.H.); 3 Shenzhen Key Laboratory of Marine Genomics, Guangdong Provincial Key Lab of Molecular Breeding in Marine Economic Animals, Shenzhen 518083, China; lijia1@genomics.cn (J.L.); bianchao@genomics.cn (C.B.); youxinxin@genomics.cn (X.Y.); 4 Nanjing Institute of Fisheries Science, Nanjing 210029, China; biowliu@163.com; 5 BGI Zhenjiang Institute of Hydrobiology, Zhenjiang 212000, China; guruobo@genomics.cn (R.G.); xujunmin@genomics.cn (J.X.); 6 School of Veterinary Medicine, Rakuno Gakuen University, Ebetsu 069-8501, Japan

**Keywords:** Chinese yellow catfish, whole genome sequencing, toxin genes, identification

## Abstract

Naturally derived toxins from animals are good raw materials for drug development. As a representative venomous teleost, Chinese yellow catfish (*Pelteobagrus fulvidraco*) can provide valuable resources for studies on toxin genes. Its venom glands are located in the pectoral and dorsal fins. Although with such interesting biologic traits and great value in economy, Chinese yellow catfish is still lacking a sequenced genome. Here, we report a high-quality genome assembly of Chinese yellow catfish using a combination of next-generation Illumina and third-generation PacBio sequencing platforms. The final assembly reached 714 Mb, with a contig N50 of 970 kb and a scaffold N50 of 3.65 Mb, respectively. We also annotated 21,562 protein-coding genes, in which 97.59% were assigned at least one functional annotation. Based on the genome sequence, we analyzed toxin genes in Chinese yellow catfish. Finally, we identified 207 toxin genes and classified them into three major groups. Interestingly, we also expanded a previously reported sex-related region (to ≈6 Mb) in the achieved genome assembly, and localized two important toxin genes within this region. In summary, we assembled a high-quality genome of Chinese yellow catfish and performed high-throughput identification of toxin genes from a genomic view. Therefore, the limited number of toxin sequences in public databases will be remarkably improved once we integrate multi-omics data from more and more sequenced species.

## 1. Introduction

As one venomous bony fish in the order of Siluriformes, Chinese yellow catfish (*Pelteobagrus fulvidraco*) has been an economically important freshwater species in China because of its good meat quality [1]. In 2016, the Chinese yellow catfish production in China was over 300,000 tons with an elevation of 20% from the previous year [2]. In our previous study [3], we reported a novel multi-omics pipeline to predict toxin genes from the venom glands of Chinese yellow catfish based on transcriptomic and proteomic sequencing. Here, we performed whole genome sequencing of this venomous teleost to provide another valuable genetic resource for high-throughput identification of toxin genes.

As we discussed before [3], aquatic venoms have been largely ignored as a resource for potential pharmaceuticals, although there are more aquatic venomous species than the total of venomous terrestrial animals [3]. The limited number of toxin sequences [3,4] has been an obstacle for development of novel marine drugs.

Whole genome resources provide excellent templates and genetic bases for further exploration of toxin genes. Over the past decades, toxin genes have attracted much attention due to their functionality and evolutionary genesis in various species [5]. Recent studies have discovered the accelerated evolution in snake venom toxin genes, which was indicated by the exonization and intronization of disintegrin or metalloprotease genes [6]. Prey-specific toxin genes, *sulditoxin* and *sulmotoxin* 1, also exhibit neofunctionalization and rapidly adaptive evolution [7]. Adaptive evolution of animal toxin multigene families at the intraspecies and interspecies levels had also been investigated [8]. Thus, it is worth investigating the complex venom systems, especially in this “omics” era [9]. As only a few venomous fish genomes are available, while teleost comprises a large part of the world vertebrates, we have been anxious to systematically enrich findings of toxin genes and expedite our understanding of venoms in teleost.

In our present study, we not only generated a high-quality genome assembly of the Chinese yellow catfish, but also established an integrated strategy to identify toxin genes from a genomic view. It seems to be an effective way to increase the number of toxin sequences, which will be very useful for rapid development of novel marine drugs. On the other hand, the whole genome sequence will also be beneficial to further molecular breeding of this economically important fish.

## 2. Results

### 2.1. Summary of Sequencing Data and Genome-size Estimation

A total of 314.37 gigabases (Gb) of raw reads were generated in a next-generation Illumina (San Diego, CA, USA) sequencing platform (Table S1; see more details in Section 5.1). After employing SOAPfilter v2.2 (http://soap.genomics.org.cn/index.html) to remove low-quality reads as well as PCR-replicates and adapter sequences, we obtained 231.60 Gb of clean data for subsequent assembling. Meanwhile, in order to improve the assembly quality with third-generation sequencing, we also acquired 25.47 Gb of sequencing data in a PacBio (Pacific Biosciences, Menlo Park, CA, USA) sequencing platform, with an average length of 7.10 kb (Table S2).

**Figure 1:**
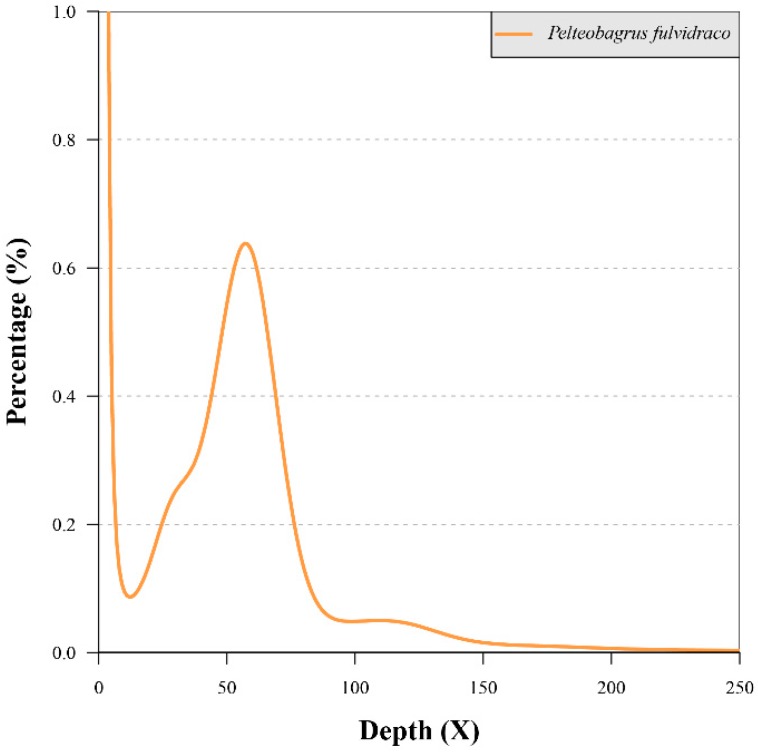
The 17-mer distribution of Chinese yellow catfish. Sequencing data from the Illumina short-insert libraries (200, 500, and 800 bp) were used for this analysis. The x-axis is the sequencing depth of each unique 17-mer, and the y-axis is the percentage of unique 17-mers. The peak depth was 57, and the percentage for peak (0.638%) was based on the total k-mer number (410,049,532,138).

Based on our achieved 17-mer distribution (Figure 1), we determined that the total k-mer number and k-mer depth was 410,049,532,138 and 57 respectively. Therefore, we estimated that the genome size of Chinese yellow catfish is 720 Mb (Table S3 and Figure 1; see more details about the calculation in Section 5.2).

### 2.2. Generation of a High-quality Whole-genome Assembly

#### 2.2.1. Primary De Novo Genome Assembly

We performed a hybrid strategy to generate a primary de novo genome assembly. First, Platanus v1.2.4 (Tokyo Institute of Technology, Tokyo, Japan) [10] with an optimized parameter “−k 35” was employed to obtain a De Bruijin graph assembly by using Illumina short-insert reads. We generated a total of 2,880,541 contigs, with the length of contig N50 at 1054 bp. Subsequently, we employed the DBG2OLC [11] program to align these contigs upon the PacBio reads for construction of consensus contigs. Finally, we used Pilon v1.22 (Broad Institute of MIT and Harvard, Cambridge, MA, USA) [12] to polish the assembly. As a result, we assembled a genome with the total size of 703 Mb and the length of contig N50 at 705 kb.

#### 2.2.2. Genome Scaffolding

Based on the primary assembly of contigs, we collected PacBio reads to construct scaffolds by using SSPACE-LongRead (Genome Analysis and Technology Department, Leiden University, Leiden, The Netherlands) [13]. After that, we employed Illumina long-insert libraries (2, 5, 10, and 20 kb) to operate scaffolding again by performing SSPACE_Standard [14]. We then used GapCloser (BGI, Shenzhen, China) [15], GapFiller [16] and PBjelly (Baylor College of Medicine, Houston, TX, USA) [17] to fill the gaps of each scaffold. We subsequently applied Pilon v1.22 again to finish the last round of polishing. Finally, we generated a 714-Mb genome (99.17% of the estimated genome size), with 663 scaffolds, a scaffold N50 of 3.65 Mb and a contig N50 of 970 Kb (see more details in Table 1). 

#### 2.2.3. Evaluation of the Achieved Genome Assembly

**Figure 2:**
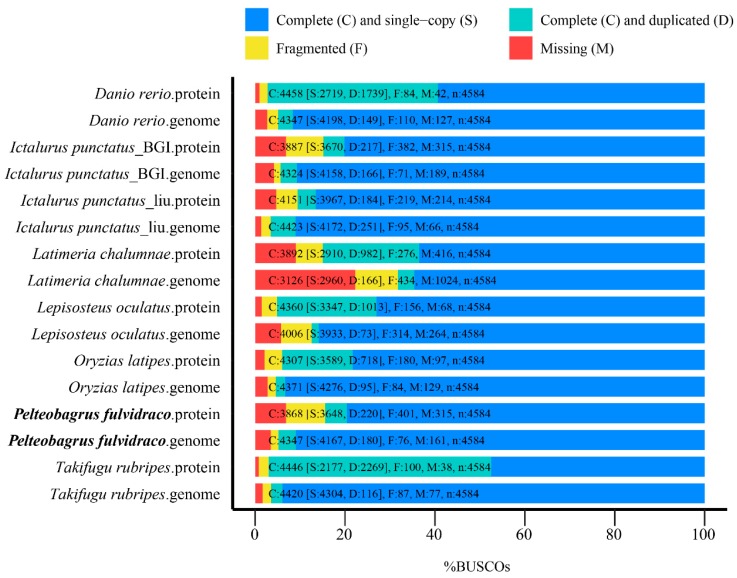
The BUSCO assessment of genomes from Chinese yellow catfish and other fish species. The genome-level benchmarking value of Chinese yellow catfish was C: 94.8% (containing S: 90.9%, D: 3.9%, F: 1.7%, M: 3.5%, *n*: 4584), and the corresponding protein-level benchmarking value was C: 84.4% (including S: 79.6%, D: 4.8%, F: 8.7%, M: 6.9%, *n*: 4584). Abbreviations: C, complete; S, Complete and single-copy; D, duplicated; F, fragmental; M, missed; n: total BUSCO groups for searching.

After the polishing procedures, we employed BUSCO (University of Geneva Medical School and Swiss Institute of Bioinformatics, Geneva, Switzerland) [18] to evaluate the completeness of our assembly. The actinopterygii_odb9 [19] orthologues gene set was used as the BUSCO reference. Our results demonstrated that the genome-level benchmarking value was 94.8%, containing S: 90.9%, D: 3.9%, F: 1.7%, M: 3.5%, *n*: 4584 (S: complete and single-copy, D: complete and duplicated, F: fragmental, M: missed, *n*: total BUSCO groups for searching). The comparative BUSCO data indicate high-quality of our assembled coverage (Figure 2).

Meanwhile, we employed the available transcriptomic data (see more details in Section 5.3.2) to validate the genome coverage. The de novo assembled transcripts were re-aligned to the genome assembly, and the results demonstrated that our genome assembly covered over 98% of gene regions (the middle column in Table 2). These data also confirmed the high level of completeness and accuracy of our genome assembly.

Additionally, the variation of GC content in Chinese yellow catfish was calculated with 50-kb non-overlapping sliding windows. Our result depicted that the observed GC content showed no sequencing-based GC preference (Figures S1 and S2), suggesting a good purity of our generated assembly (without contamination of prokaryotes).

### 2.3. Genome Annotation

Repetitive sequences accounted for 33.99% of the whole genome assembly. A detailed proportion of the predominant families of repetitive sequences is summarized in Table S4.

A total of 21,562 genes with an average of 9.46 exons and 1698-bp coding-region of each gene were predicted (see more details in Table S5). After the routine functional annotation, we predicted that 97.59% genes were with at least one related functional assignment (Table S6). Similarly, BUSCO was also used to assess the completeness of Chinese yellow catfish gene set, and a protein-level benchmarking value of 84.4% (Figure 2) was achieved.

### 2.4. Phylogenetic Analysis and Divergence-Time Estimation of Chinese Yellow Catfish

**Figure 3:**
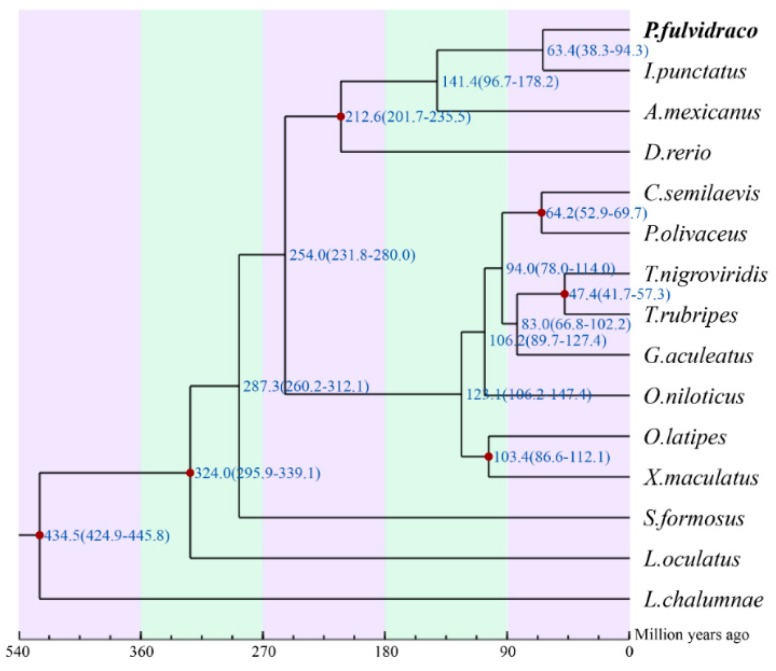
The phylogenetic tree of yellow catfish and other 14 related fish species. The red dot nodes have been validated based on the TimeTree (http://www.timetree.org/). Numbers represent the estimated divergence times.

In the present study, we obtained 1156 one-to-one orthologous genes among Chinese yellow catfish and other 14 examined teleost species (find more details of species names in Figure 3, Table S7 and Section 5.2). Our final phylogenetic analysis indicates that the divergence time of the Chinese yellow catfish and the nearest channel catfish (*Ictalurus punctatus*) was 63.4 million years ago (mya), with a confidence interval of 38.3–94.3 mya (the numbers at top of Figure 3).

### 2.5. High-Throughput Identification of Toxin Genes

Based on our previous report of toxin genes from Chinese yellow catfish transcriptomes [4], we identified 37 toxin genes in the yellow catfish. However, based on the 6665 reference toxin genes that were collected from NCBI [3,4], we obtained 202 toxin genes from our genome assembly. After removal of low-quality sequences, we finally constructed a local non-redundant database with 207 toxin genes for the Chinese yellow catfish.

On the basis of translated amino acids (aa) of each gene, we manually divided these toxin genes into three groups, including the short-length group (less than 100 aa), the medium-length group (between 100 and 300 aa), and the long-length group (over 300 aa). Finally, we determined that these three groups included 125, 61, and 21 toxins genes, respectively. Related protein sequences are provided in Data S1–S3.

#### 2.5.1. The Short-Length Toxin Genes

The 125 genes with an entire length less than 100 aa (Data S1) accounted for the vast majority of Chinese yellow catfish toxin genes. After alignment searching of public databases, we found that these genes were annotated as “fragmental,” which means these genes do not have full structures. However, the typical motif of venom proteins, “Gly-X-Cys (X means any other amino acids),” existed in most of these genes (80/125). Meanwhile, 90.4% (113/125) of these genes contained at least one cysteine. Usually, the number of cysteine in one single toxin gene varied from 1 to 15.

#### 2.5.2. The Medium-Length Toxin Genes

**Figure 4:**
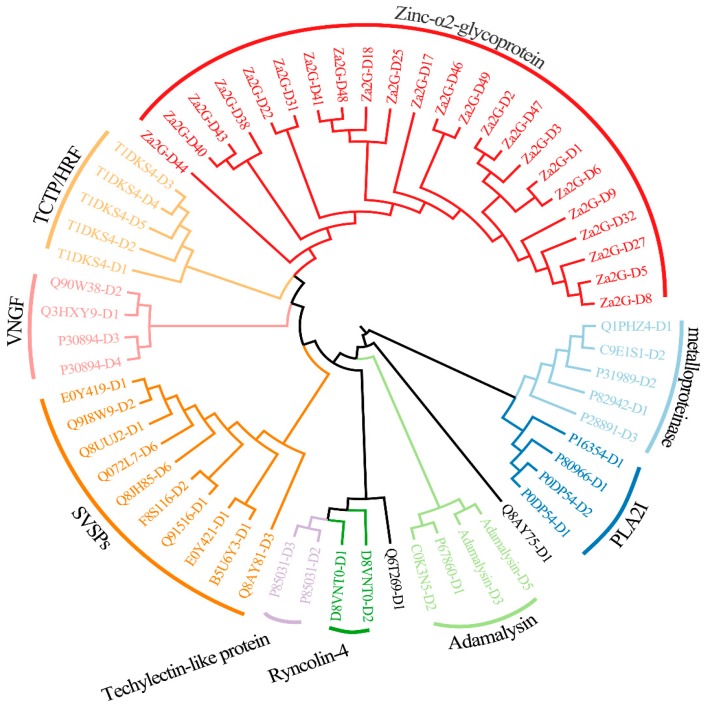
A phylogenetic classification of the nine subgroups of medium-length toxin genes. Two other genes “Q6T269-D1” and “Q8AY75-D1,” however, do not belong to any subgroup.

Each gene within this group contained at least one copy of cysteine, and the maximal number of cysteine in *Zinc metalloproteinase-disintegrin-like* gene reached 26. In the 61 medium-length toxin genes (Data S2), 59 had been attributed into nine subgroups (Figure 4) on the basis of differences in sequences and secondary structures. The detailed information of each subgroup was summarized as follows.

(1) Twenty-three toxin genes (37.70%) can code Zinc-α2-glycoprotein (Za2G), which may play significant roles in prohibiting growth and proliferation of tumors [20].

(2) Ten toxin genes are identified as snake venom serine proteinase (SVSPs) [21]. It was reported that cascade SVSPs disturb hemostasis by acting on related proteins in blood coagulation [22]. 

(3) Four toxin genes are adamalysin, which were firstly reported in Eastern diamond back rattlesnake [23], although their functions are still unknown.

(4) Five genes are classified as venom metalloproteinases, which belong to the metzincin family and typically show extracellular hemorrhagic activity [24]. Venom metalloproteinases are vastly involved in the local and systemic hemorrhage, such as reducing blood supply, leading to ischemia and causing damage to microvasculature [25].

(5) Five genes decode translationally controlled tumor proteins (TCTP/HRF), which are recognized as venom toxins in different genera of spiders and snakes [26].

(6) Four genes are categorized into the phospholipase A2 family. The most important Phospholipase A2 I (PLA2I) plays a myotoxic role in a venomous pitviper (*Porthidium lansbergii lansbergii*) [27].

(7) Four genes are annotated as venom nerve growth factor (VNGF). VNGF belongs to the neutrotrophin family, which plays an important role in the survival of neuronal cells [28].

(8) Two genes encode a techylectin-like protein. The basic information of the techylectin-like protein was previously reported in a spider (*Phoneutria nigriventer*) [29].

(9) Two genes encode ryncolin-4, which was primarily predicted in the reef-building coral (*Acropora digitifera*) through a venom proteomic expression profiling analysis [30].

The remainder two toxin genes, “Q6T269-D1” and “Q8AY75-D1,” did not belong to the above-mentioned subgroups. After searching public databases, we annotated Q6T269-D1 as a Kunitz-type serine protease inhibitor and Q8AY75-D1 as a Calglandulin protein.

#### 2.5.3. The Long-Length Toxin Genes

**Figure 5:**
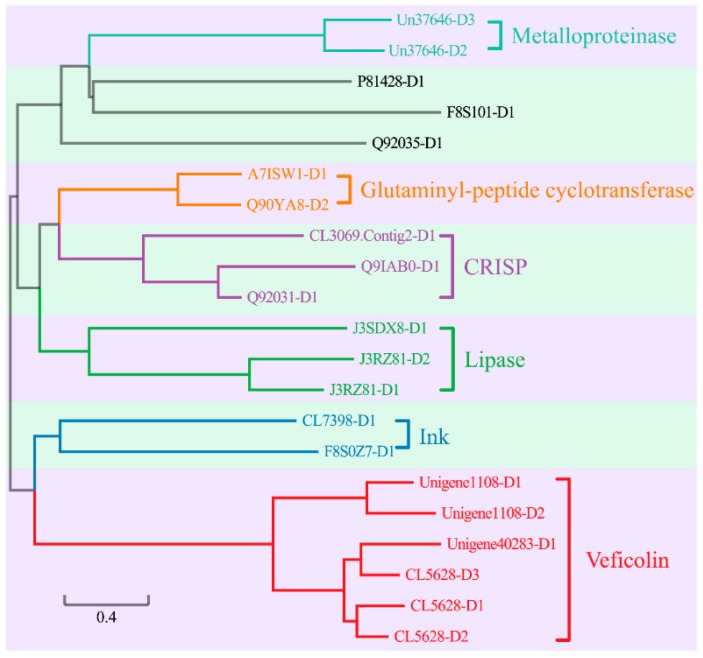
A phylogenetic classification of the 21 long-length toxin genes. Three other genes “P81428-D1,” “F8S101-D1,” and “Q92035-D1,” do not belong to the classified six families.

In this group, the maximal number of cysteine in one gene is up to 69. Among the 21 toxin genes (Data S3), 18 are assigned into six families (Figure 5). More specifically, two genes are annotated as the venom metalloproteinases. Two genes belong to glutaminyl-peptide cyclotransferase family, which has been identified from the venoms of the Taiwanese snake [31] and bumblebee [32]. Three genes are in the cysteine-rich secretory protein (CRISP) family. Recent studies had uncovered that CRISPs are widely distributing in snake venoms. The main functions of CRISPs include prohibition of smooth muscle contraction and closure of nucleotide-gated ion channels leading to lethargy, hypothermia, and paralysis [33]. Three genes belong to the lipase family. This family had been previously isolated from anguimorph lizard venoms [34]. However, by far the functional study about lipase family is scarce. Two genes are thought to be in the ink toxin family, which had been firstly extracted from purple ink secretions of sea hares. They had been proven to have positive effects in antimicrobial and antitumor studies [35]. Five toxin genes belong to the veficolin family. The main feature of veficolin is the G-X-Y repeats (Glycine plus two other amino acids). By far, veficolins had been predicted to be involved in constriction of platelet aggregation [36].

### 2.6. Identification of Toxin Genes in a Special Sex-Related Region

**Figure 6:**
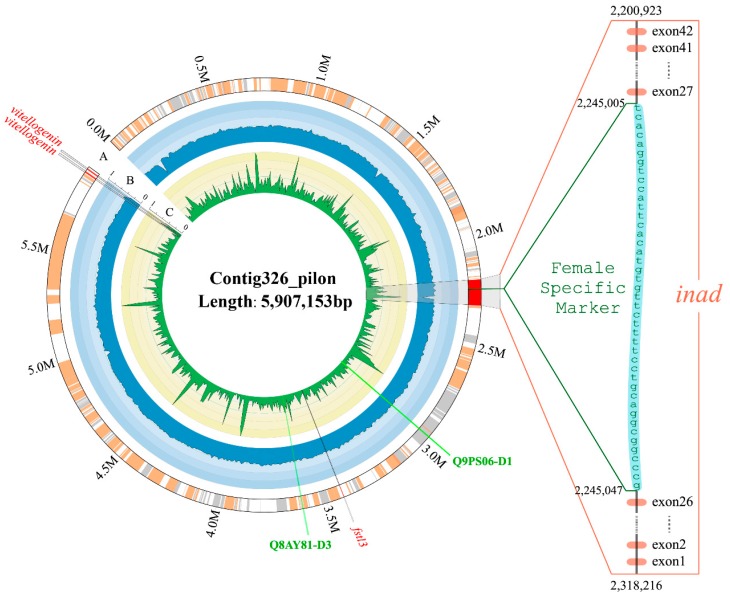
Distribution of a female specific marker and other sex-related/toxin genes in the Contig326_pilon. Two toxin gens, “Q9PS06-D1” and “Q8AY81-D3,” were presented in dark green. The female specific marker, located in the intron26 of *inad* gene, was 42 bp in length. The left circos atlas represents the entire Contig326_pilon. Its rings from outside to inside include: (A) nucleotide sequence of the Contig326_pilon, (B) percentage of GC content in 10-kb non-overlapping windows, and (C) percentage of repeat elements in 10-kb non-overlapping windows. In the Contig326_pilon, faint yellow ribbons represent “+” orientating genes, while grey ribbons represent “−” orientating genes; sex-related genes and *inad* were drawn with a red ribbon.

In the present study, two previously published sex-specific sequences and eleven markers (see more details in Section 5.6) were searched against our achieved genome assembly. Only one female-specific marker mapped one time with 100% alignment rate in a 6-Mb region (Contig326_pilon; Figure 6). More specifically, this marker located in the intronic region of *inad* (inaD-like protein) gene. The main function of *inad* is to mediate protein-protein interactions, which had been validated in previous studies [37].

Interestingly, we also identified two toxin genes in this special sex-related region. They are snaclec coagulation factor IX/factor X-binding protein subunit B (Q9PS06-D1) and thrombin-like enzyme (Q8AY81-D3), respectively. The former, localized at ≈2.95 Mb of the Contig326_pilon and previously reported in a venomous viper (*Echiscarinatus carinatus*) [38], can combine with anticoagulant factor IX and factor X to form an anticoagulant protein [39]; the latter, belonging to the SVSP family (Figure 4) and localized at ≈3.6 Mb of the Contig326_pilon, may cause various pathological effects, such as disturbance in the hemostatic system, platelet aggregation, neurologic disorders, thrombosis, and activation of coagulation factors [40].

## 3. Discussion

### 3.1. A Good Strategy to Generate the High-Quality Genome Assembly

With the rapid development of next-generation sequencing (NGS) technology, the output of sequencing platforms has risen vastly, whilst the price per Gb of data are dropping quickly. These advances allow researchers to easily decode the whole genome sequences. With the help of NGS, many fish genomes have been reported, such as Atlantic herring [41], channel catfish [42], mudskippers [43], half-smooth tongue sole [44], large yellow croaker [45], and so on.

In recent years, third-generation sequencing technology, also recognized as long-read sequencing, has been soaring. In comparison with NGS and first-generation sequencing technologies, third-generation sequencing has the distinct advantage of the length of sequencing reads, i.e., production of much longer reads than NGS. However, the sequencing errors happen at random in third-generation sequencing, which means we can dramatically reduce the sequencing errors through increasing the sequencing depth. The sequencing errors of NGS in Illumina sequencing platforms [46], by contrast, possibly increase because of either sequence-specific alterations in enzyme preference or single-strand DNA folding. Third-generation sequencing with longer read length will effectively alleviate tremendous computing workload for genome assembly. Nowadays, more and more fish genomes have been sequenced by using third-generation sequencing, such as Asian seabass [47] and Chinese sillago [48]. Collectively, these fish genome sequences will promote the biological research and molecular breeding of these interesting fishes.

As a representative venomous freshwater fish with high economic value, Chinese yellow catfish is extensively available in river basins of China, such as the Yangtze River and Huaihe River. Since the nutrient-rich flesh of Chinese yellow catfish have a high elasticity and pleasant firmness [49], the artificial culture of Chinese yellow catfish has been highly recognized. Although the scale of its aquaculture industry has been expanding, the wild germplasm resources of Chinese yellow catfish have degenerated continually because of overfishing and habitat contamination. Traditional breeding methods are too time-consuming to support the rapid development of its industry. However, genome-based marker-assisted breeding will become a more efficient and holistic approach after realization of whole genome sequencing [50,51]. In this study, we also employed a PacBio sequencing platform to yield long sequencing reads for a high-quality assembly. The PacBio SMAT (Single-Molecular Real-Time sequencing) is a typical third-generation sequencing platform and has been widely used for whole genome sequencing. At the same time, we also adopt the Illumina NGS sequencing platform to create the short sequencing reads. With the hybrid assembling of these two different types of reads, we decoded the Chinese yellow catfish genome with a high quality based on the BUSCO assessments.

The availability of Chinese yellow catfish whole genome can narrow down the gaps between genotypes and phenotypes. We here provided a good reference genome for further biological studies and molecular breeding of this economically important teleost.

### 3.2. High Efficiency to Identify Toxin Sequences

Acquisition of toxin genes through sequencing technology had been proven to be efficient and practicable. Based on transcriptomes, we have identified conotoxins from cone snails in a high-throughput way. In the Chinese tubular cone snail (*Conus betulinus*) [52,53], we identified a total of 215 distinct conotoxins, in which 183 are novel.

Chinese yellow catfish, sometimes called stringing catfish in South China, has venom glands in the sharp spines of the dorsal and pectoral fins. Venom is produced by glandular cells in the epidermal tissue of the spines [3,4]. The symptoms of yellow catfish venom intoxication include local pain, edema, bleeding, and even serious and painful injuries to human [3,54]. However, the previous studies of Chinese yellow catfish venom were generally based on molecular markers or transcriptomic analyses [4]. Although more than two dozen toxin genes had been reported in Chinese yellow catfish, we still lack comprehensive understanding about the venom. The deciphering of Chinese yellow catfish genome can provide a fundamental genetic resource for venom studies.

In our present research, based on the whole genome sequences, we constructed a local venom database of Chinese yellow catfish. With one shot, we obtained 207 toxin sequences. The outcome also depicted that a complex structural differentiation of venoms may exist in Chinese yellow catfish, which was supported by our previous transcriptome study [3,4].

There is no denying that the fragmental toxin genes occupy a considerable proportion in the annotated toxin genes. We assumed two possible reasons to explain the results. The first is limitation of the reference databases of toxin genes. There are limited species that have been reported with toxins, and the main study objects focused on snakes, spiders, and a few invertebrates. Only few researches on fish toxin genes have been published by far. The lacking reference information of toxins constricts the deep research of toxin genes in many fishes including the Chinese yellow catfish. The second reason is the constraints of currently available technologies. Nowadays, molecular and pharmacological methods are still the traditional ways to investigate functions of toxins. However, they are time-consuming and inefficient. Fortunately, with the advance of genome and transcriptome sequencing techniques, more and more genomic sequences will be available for toxin discovery and drug development.

## 4. Conclusions

We performed whole-genome sequencing, using a combination of traditional next-generation and new third-generation sequencing strategies, to generate a high-quality genome reference for Chinese yellow catfish. Based on the achieved genome assembly, we identified 207 toxin genes in a high-throughput way. We also preliminarily classified these toxin genes into three main groups on the basis of their protein sequence length. In summary, we provide a valuable genetic resource for high-throughput identification of toxin genes in the venomous yellow catfish. These toxin genes will be useful for further development of drugs and pesticides.

## 5. Materials and Methods

### 5.1. Sampling and Genome Sequencing

To generate genome sequencing data of Chinese yellow catfish, we adopted two different strategies. The first one was the traditional Illumina whole-genome sequencing strategy. The detailed procedures were provided in the followed sections. Genomic DNAs were isolated from muscle tissue of a female Chinese yellow catfish, which was collected from a fish farm in Jiangsu province, China. Seven paired-end sequencing libraries, including three short-insert libraries (200, 500, and 800 bp) and four long-insert libraries (2, 5, 10, and 20 kb), were constructed using the standard operating protocol provided by Illumina (San Diego, CA, USA). Finally, paired-end sequencing was performed using the Illumina HiSeq X-Ten platform. The second way was the PacBio single-molecule real-time sequencing strategy. Kidney genomic DNA was extracted for the construction of a 20-kb insert-size library, which was sequenced in a PacBio Bioscience Sequel platform. 

All the animal experiments were approved by the Institutional Review Board on Bioethics and Biosafety of BGI (No. FT1510).

### 5.2. Estimation of Genome Size

Generally speaking, the distribution of k-mers is subjected to a Poisson distribution [55]. In this study, we estimated the genome size of Chinese yellow catfish using the k-mer method and the following equation (Equation (1)):

G = k-mer_number/k-mer_depth(1)

where the G is the genome size, the k-mer_number is the total number of k-mers, and k-mer_depth means the peak frequency of k-mer analysis.

### 5.3. Genome Annotation

#### 5.3.1. Repeat Annotation

We applied two different methods to annotate the repeat elements of Chinese yellow catfish genome. The first method was de novo prediction. Software including RepeatModeller v1.08 (http://www.repeatmasker.org/RepeatModeler/) and LTR_FINDER v1.0.6 (Fudan University, Shanghai, China) [56] was employed to generate the local repeat reference. Subsequently, the achieved genome sequences were aligned against this reference to produce the de novo predicted repeat elements. The second method was the homology-based prediction. Our assembly was aligned to the RepBase v21.01 (Genetic Information Research Institute, Sunnyvale, CA, USA) [57] by using RepeatMasker v4.06 and RepeatProteinMask v4.06 (Institute for Systems Biology, Seattle, WA, USA) [58]. Finally, the data from two methods were integrated to generate the non-redundant results.

#### 5.3.2. Annotation of Gene Set

We utilized three different strategies to annotate the whole gene set. The first strategy was ab initio annotation. After masking the genomic repetitive elements, AUGUSTUS v2.5 (Institute of Microbiology and Genetics, University of Göttingen, Göttingen, Germany) [59] and GENSCAN v1.0 (Stanford University, Stanford, CA, USA) [60] were employed to ab initio predict genes. The second method was homologous-gene-based annotation. We firstly downloaded the protein sequences of zebrafish (*Danio rerio*), Atlantic cod (*Gadus morhua*), coelacanth (*Latimeria chalumnae*), medaka (*Oryzias latipes*), Japanese puffer (*Takifugu rubripes*), pufferfish (*Tetraodon nigroviridis*), Nile tilapia (*Oreochromis niloticus*), platyfish (*Xiphophorus maculatus*), and three-spined stickleback (*Gasterosteus aculeatus*) from the Ensembl database (release version 87). These protein sequences were used to search for best-hit alignments in the generated yellow catfish genome using the Tblastn (National Center for Biotechnology Information, Bethesda, MD, USA) [61] program, with the channel catfish proteins [42] as the reference. Subsequently, GeneWise v2.2.0 (The European Bioinformatics Institute, Cambridge, UK) [62] was employed to identify the potential gene structure of each best-hit alignment. The third method was the transcriptome-based prediction. We used two different transcriptomic data, including our previously reported data [4] and the muscle transcriptomic data sequenced by an Illumina platform. Tophat v2.1.1 (Johns Hopkins University, Baltimore, MD, USA) [63] and Cufflinks v2.2.1 (http://cufflinks.cbcb.umd.edu/) were performed to generate the whole gene set. Finally, GLEAN (Texas A & M University, College Station, TX, USA) [64] was utilized to produce the consensus results by integration of the data from above-mentioned three methods.

The predicted genes of Chinese yellow catfish were used to search several public functional databases, including NCBI-Nr (non-redundant protein sequences), Swiss-Prot [65], Interpro [66], TrEMBL, and KEGG [67], for identification of functional motifs and domains by using BLAST (National Center for Biotechnology Information, Bethesda, MD, USA).

### 5.4. Phylogenetic Analysis

We downloaded the proteomes of 14 species from public databases (Table S7). These proteomes contained a total of 316,447 proteins. The one-to-one orthologous proteins were generated by using Blastp [61] and Hcluster_sg [68]. First, Blastp was performed to generate the best-hit for each protein. Then, Hcluster_sg with parameter setting of “-w 10 -s 0.34” was used to identify the one-to-one orthologous proteins among these species.

Subsequently, MrMTgui program was employed to obtain the best nucleotide substitution model (“GTR + I + G”). Based on the best substitution model, MrBayes v3.1.2 (Swedish Museum of Natural History, Stockholm, Sweden) [69] with generation setting to 1,000,000 was performed to construct the phylogenetic trees. Mcmctree (PAML package) [70] was operated to estimate divergence times.

### 5.5. Prediction of Toxin Genes

Fifteen putative toxin genes had been consolidated based on transcriptomic and proteomic data in our previous study [4]. In the present study, the following two ways were combined to predict toxin genes in the yellow catfish. First, we searched the 15 putative toxin genes in the yellow catfish genome using the Blastp program. Second, a reference database including 6665 toxin genes was used to search against the Chinese yellow catfish genome. The reference database was generated by the following methods: (1) toxin sequences were downloaded from NCBI by using keywords “Toxin” and “Venom,” and (2) these sequences were realigned with the NCBI-Nr database for validation. Finally, we merged the results from the two ways and filtered those genes with premature termination or low alignment rates (lower than 50%).

### 5.6. Localization of Potential Toxin Genes in the Sex-Related Region

In previous studies [51,71], the sex-determination of Chinese yellow catfish was reported as the XY system. Two fragmental sequences had been proven to be associated with sex-specificity, including one 8102-bp male-specific sequence and another 5362-bp female-specific sequence. Meanwhile, three male-specific markers and eight female-specific makers have been identified before [72]. In our current study, these sequences were downloaded from NCBI, and they were aligned against the yellow catfish genome assembly by using the Blastn program. We filtered the results with a threshed of 100% match and one hit.

## Figures and Tables

**Table 1:** Summary of the assembled genome in each procedure.

Step	Software	Contig N50 (bp)	Maximum Contig (bp)	Minimum Contig (bp)	Scaffold N50 (bp)	Maximum Scaffold (bp)	Minimum Scaffold (bp)	Total Size (bp)
Contig assembling	Platanus	1054	49,678	109	-	-	-	1,010,987,672
	DBG2OLC	707,335	6,076,047	268	-	-	-	706,928,086
Polishing round 1	Pilon	705,180	6,050,085	270	-	-	-	702,622,905
Scaffolding	SSPACELongRead	982,636	6,050,085	270	1,109,190	7,365,535	270	706,306,982
	SSPACE_Standard	705,180	6,050,085	270	3,655,204	19,552,289	270	712,893,760
Gap filling	Gapcloser	813,785	11,966,130	270	3,655,204	19,552,617	270	712,834,712
	GapFiller	859,168	11,966,116	270	3,655,204	19,552,752	270	712,901,309
	PBjelly	962,661	14,953,314	270	3,655,300	19,560,773	270	714,800,876
Polishing round 2	Pilon	970,098	15,455,883	277	3,653,474	19,544,699	277	713,824,612

**Table 2:** Evaluation the completeness of gene regions in our genome assembly by assembled transcripts.

Dataset	Number of EST Clusters	Total Length (bp)	Coverage Rate by the Assembly (%)	with >90% Sequence in One Scaffold		with >50% Sequence in One Scaffold	
				Number	Percentage (%)	Number	Percentage (%)
>0 bp	78,225	57,694,186	98.1907917	73,167	93.53404	77,222	98.7178
>200 bp	60,258	54,613,314	98.2312921	56,311	93.44983	59,575	98.86654
>500 bp	30,229	45,487,954	98.32383756	28,117	93.01333	29,963	99.12005
>1000 bp	17,675	36,547,853	98.41627906	16,434	92.97878	17,543	99.25318

## Supplementary Materials

The following materials are available online at http://www.mdpi.com/2072-6651/10/12/488/s1. Figure S1: GC content and sequencing depth of the yellow catfish genome. Figure S2: Comparisons of GC content between the yellow catfish and other seven fish species. Table S1: Summary of the next-generation sequencing data from an Illumina X-Ten platform. Table S2: Summary of the third-generation sequencing data from a PacBio Bioscience Sequel platform. Table S3: Genome-size estimation based on the 17-mer frequencies. Table S4: The detailed repetitive elements in the yellow catfish genome. Table S5: Statistics of gene annotation from the genome assembly of Chinese yellow catfish. Table S6: Functional assignments from the genome assembly of Chinese yellow catfish. Table S7: Information of the fish species used for phylogenetic analyses. Data S1: The short-length toxin proteins in the yellow catfish. Data S2: The medium-length toxin proteins in the yellow catfish. Data S3: The long-length toxin proteins in the yellow catfish. Data Availability: The genome assembly of Chinese yellow catfish has been deposited at the NCBI Genbank under the project ID of PRJNA494039.
