# Supplementary material for: Whole-Genome Sequencing of Chinese Yellow Catfish Provides a Valuable Genetic Resource for High-Throughput Identification of Toxin Genes
Source: Toxins (Basel). 2018 Nov 23;10(12):488. doi: 10.3390/toxins10120488 (PMC6316204; doi:10.3390/toxins10120488)
Supplement: Supplementary file 1 [file toxins-10-00488-s001.pdf]

Supplementary Materials: Whole-Genome Sequencing of Chinese Yellow Catfish Provides a Valuable Genetic Resource for High-Throughput Identification of Toxin Genes

Shiyong Zhang, Jia Li, Qin Qin, Wei Liu, Chao Bian, Yunhai Yi, Minghua Wang, Liqiang Zhong, Xinxin You, Shengkai Tang, Yanshan Liu, Yu Huang, Ruobo Gu, Junmin Xu, Wenji Bian, Qiong Shi, and Xiaohui Chen

Data Availability: The genome assembly of Chinese yellow catfish has been deposited at the NCBI Genbank under the project ID of PRJNA494039.

Table S1. Summary of the next-generation sequencing data from an Illumina X-Ten platform.

Insert Size (bp)	Sequencing Length (bp)	Raw Data (Gb)	Clean Data (Gb)
250	150	51.34	45.14
500	150	56.23	48.16
800	120	18.40	17.01
2000	150	46.08	35.62
5000	150	46.90	30.55
10,000	150	48.71	32.99
20,000	150	46.71	22.13
Total		314.37	231.60

Table S2. Summary of the third-generation sequencing data from a PacBio Bioscience Sequel platform.

Library ID	Total Bases (Gb)	Total Reads	Average Length (bp)	Max Length (bp)	Min Length (bp)	N50 (bp)
r54040_20180213_070316-2_B01	4.18	511,615	8178.57	77,479	50	13,730
r54040_20180213_070316-3_C01	4.17	534,723	7799.99	75,635	50	13,282
r54040_20180213_070316-4_D01	4.05	480,249	8435.54	66,100	50	13,944
r54040_20180213_070316-5_E01	2.08	383,891	5419.65	97,683	50	9150
r54266_20180412_092831-1_C01	6.83	1,077,808	6337.74	67,030	50	10,989
r54272_20180413_101835-2_D01	4.49	695,485	6453.19	61,481	50	11,228
Total	25.47	3,683,771	7104.11	97,683	50	11,805

Table S3. Genome-size estimation based on the 17-mer frequencies.

K-mer	K-mer Number	K-mer Depth	Genome Size	Used Base	Used Read	Sequencing Depth
17	41,049,532,138	57	720,167,230	46,390,459,050	333,807,932	64.4

Table S4. The detailed repetitive elements in the yellow catfish genome.

Type	Length	Proportion of Genome (%)
LTR	200327135	28.06
LINE	7387444	1.03
SINE	237822	0.03
DNA	33850171	4.74
Other	2584755	0.36
Unknown	5977250	0.83
Total	242600066	33.99

Table S5. Statistics of gene annotation from the genome assembly of Chinese yellow catfish.

Method	Software, Fish Species, or Data Source	Gene Number	Average Transcript Length (bp)	Average CDS Length (bp)	Average Exons Per Gene	Average Exons Length (bp)	Average Intron Length (bp)
De novo	AUGUSTUS	21,392	14,898.52	1,434.06	8.17	175.43	1,876.73
	GeneScan	28,229	16,786.98	1,478.17	7.72	191.46	2,277.95
Homolog	*Ictalurus punctatus* BGI	25,553	10,219.12	1,459.43	7.29	200.24	1,392.98
	Danio rerio	25,390	12,070.41	1,569.74	8.05	194.89	1,488.49
	Gadus morhua	22,759	8,385.83	1,197.41	6.64	180.22	1,273.59
	Gasterosteus aculeatus	26,322	7,804.13	1,171.03	6.31	185.53	1,248.77
	Latimeria chalumnae	19,518	8,582.87	1,377.42	6.99	196.92	1,201.98
	Oreochromis niloticus	24,516	8,883.31	1,324.56	7.05	187.86	1,249.24
	Oryzias latipes	26,464	7,215.32	1,148.88	6.08	189.06	1,194.92
	Takifugu rubripes	18,808	10,998.62	1,484.95	8.19	181.28	1,322.94
	Tetraodon nigroviridis	17,438	10,978.66	1,486.42	8.41	176.66	1,280.30
	Xiphophorus maculatus	23,040	9,053.33	1,353.53	7.20	188.07	1,242.49
Transcript	Dataset 1 *	20,132	9,395.39	2,147.20	8.37	256.55	983.55
	Dataset 2 **	33,361	6,890.79	1,692.43	6.05	279.97	1,030.39
Consensus		21,562	16,730.36	1,697.60	9.46	179.37	1,776.09

* Transcriptomic Dataset 1 were generated in this present work. ** Transcriptomic Dataset 2 were adopted from our previous report (Xie et al., 2016).

Table S6. Functional assignments from the genome assembly of Chinese yellow catfish.

Catagory	Gene Number	% of Gene
Total	21,562	100
Nr	20,941	97.12
Swissprot	19,647	91.12
KEGG	18,787	87.13
TrEMBL	20,936	97.10
Interpro	20,004	92.77
Annotated	21,042	97.59
Unannotated	520	2.41

Table S7. Information of the fish species used for phylogenetic analyses.

Species	Genome Version	Gene Number	Database
Astyanax mexicanus	AstMex102	23,041	ensembl
Cynoglossus semilaevis	Cse_v1.0	22,144	NCBI
Danio rerio	GRCz11	25,778	ensembl
Gasterosteus aculeatus	BROAD S1	20,785	ensembl
Ictalurus punctatus	-	21,556	BGI
Latimeria chalumnae	LatCha1	19,568	ensembl
Lepisosteus oculatus	LepOcu1	18,341	ensembl
Oreochromis niloticus	Orenil1.0	21,437	ensembl
Oryzias latipes	HdrR	19,682	ensembl
Paralichthys olivaceus	ParOli_1.1	21,787	NCBI
Scleropages formosus	-	22,274	BGI
Takifugu rubripes	FUGU 4.0	18,518	ensembl
Tetraodon nigroviridis	TETRAODON 8.0	19,595	ensembl
Xiphophorus maculatus	Xipmac4.4.2	20,379	ensembl

* The protein datasets of channel catfish (*Ictalurus punctatus*) and Asian arowana (*Scleropages formosus*) were obtained from our laboratory. All the protein datasets had been filtered as follows: 1) Only the longest transcript was retained if one gene has multiple transcripts. 2) Those protein sequences were discarded if the amino acid number was less than 20.

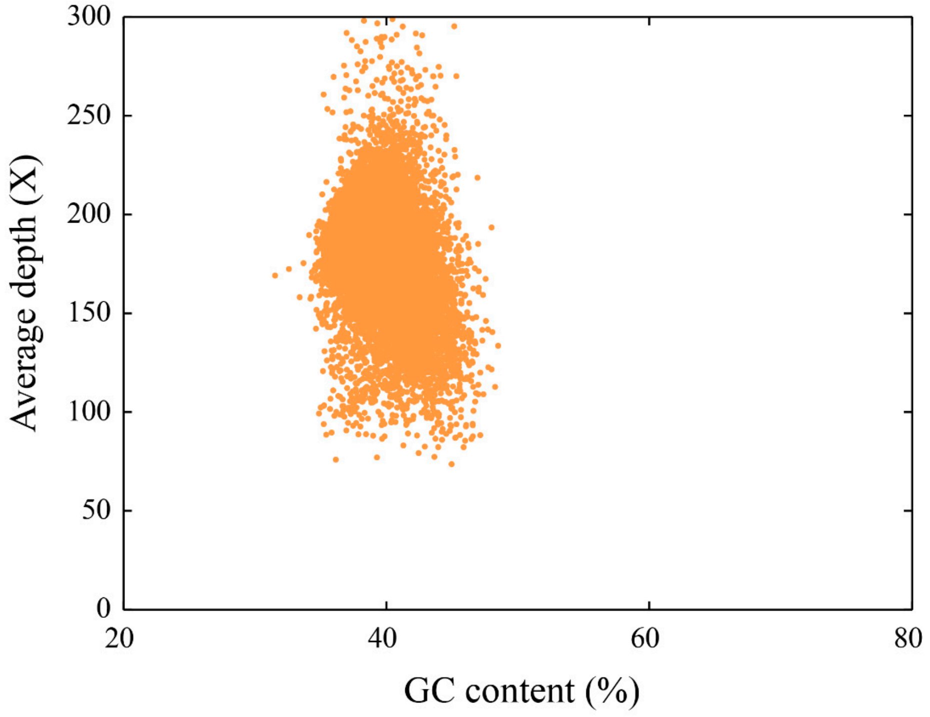

Figure S1. GC content and sequencing depth of the yellow catfish genome. This scatter plot was drawn by sliding 50-kb non-overlapping windows against the assembled yellow catfish genome. The x-axis means GC content (%), and the y-axis represents the average depth.

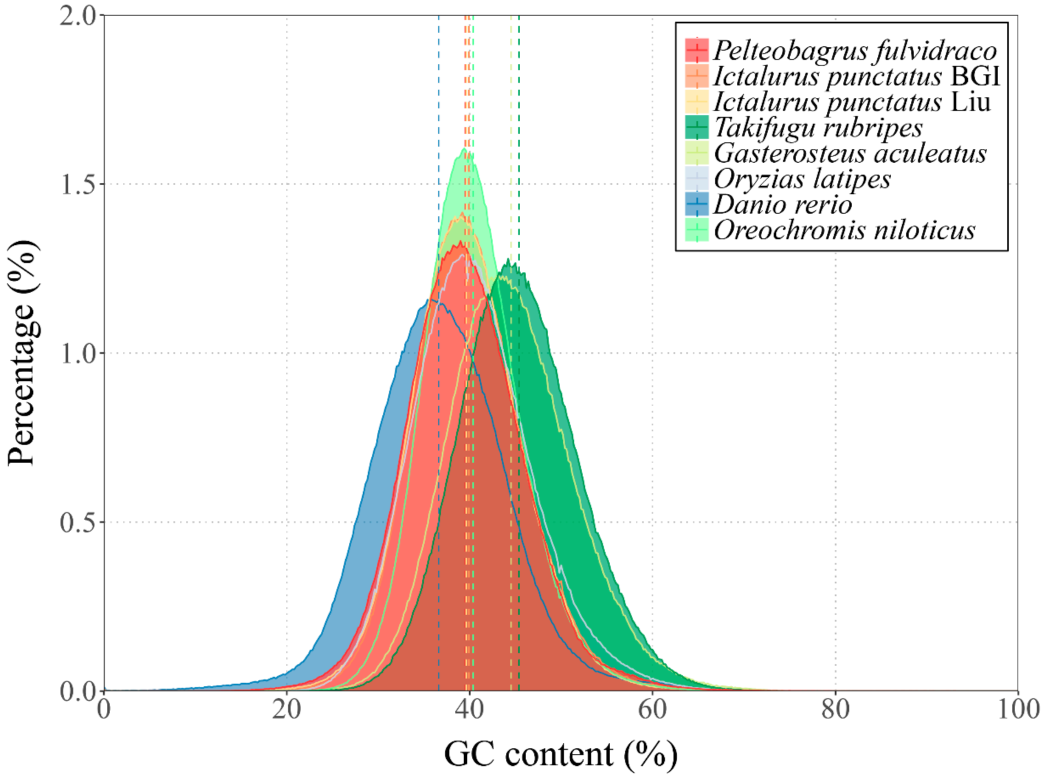

Figure S2. Comparisons of GC content between the yellow catfish and other seven fish species. The values in this plot was calculated by sliding 50-kb non-overlapping windows against these eight genome sequences. The x-axis means GC content (%), and the y-axis represents the percentage of each GC content.

Data S1. The short-length toxin proteins in the yellow catfish.

>Q4JCS0-D3
SAPYCGNKLVDVGEECDCGSEEECEKDPCCEPKTCKLRAGAECAFGVCCKYCRVTHTHT
>P0DMW6-D3
CGLPSLQGPCKFYKPRWAYSSTFNQCQPFIYGGCGGNDNNFETKEACEDLC
>P17497-D4
SMPMCGNQILEGTEECDCGPPETCNNKCCDAATCTFTKGSVCAAGVCCKDCQVLVSGTPCRVSVNECDLPEYCTGQS
GFCP
>P22028-D7
LLDPPECGNGYVEQGEECDCGS
>C0HJW4-D1
DPRSSDMNECLKKSHYYIKCFSDIC
>P82475-D1
DVYERSSCQPRETLVEVWQEFPWETHHLFLPSCVSVRRCGGCCGDEALECVPSHTDMVTMEVTPNT
>Q7Z091-D1
PCWYHSDCLCKLHKISRPSLKWLL
>P04362-D2
AWIFPGTLWCGHGNRAGDYEQLGMFERVDRCCREHDHC
>P0CI21-D24
GSARGRCEAGNGPRLAGARTSRSRVVWECSPKRVVNSI
>P0CI21-D7
GSARGRCEAGNGPRLAGARTSRSRVVWECSPKRVVNSI
>P0CI21-D8
GSARGRCEAGNGPRLAGARTSRSRVVWECSPKRVVNSI
>P81995-D2
ILDLHNKLRGQVYPPASNM
>P86468-D24
CLNGGVCHDGVKTFHCSCPKGYTGNRCQ
>P0DMW7-D4
SEVCWANAETGPCRAMLPRWYFVKEEGRCAPFIYGGCGGNRNNFESEEYCLSVC
>P86468-D5
NPCQHGGICIEKRGGYICKCTDMYTGHNCEK
>P81990-D1
QEEIVNIHNTFRREVVPTASNMLKM
>P0DN17-D8
CTQPPVTGPCRGSNTMWHYNPYEQKCSRFNYGGCQGNDNQFDTEEKCMKFC
>P86402-D1
LCVSCFTTKYDIQSTNCCGGNTCVCFG
>Q90W96-D1
CDLPHTAGLCDEWTARYYYDPASSRCVHFWYGGCPGNSNNFATMEECQQTC
>P0CV91-D2
GLFVIDDKGILRQITINDLPVGRSVDETLRLVQAFQHTDK
>P86468-D10
CLNGGTCSNTGPDKYHCSCPDGYSGQNCER
>P86468-D23
CFNGGRCADNPDGGYFCQCPTGYAGFNCEK

>P0CV91-D1
GLFIIDDKGTLRQITMNDLPVGRSVDETLRLVQAFQYTDK
>P0C8W6-D2
ASEPASEPASEPASEPASEPASEPASEPARQAARSCP
>P0DMD6-D6
CFGFKIDRISDLSGMGC
>P22028-D11
PICGNGMVEAGEQCDCG
>P0DMW6-D2
CSLPSVQGPCKSYEPRWAYNHLLKKCQSFVYGGCGGNENNFESKEACEEMC
>P00985-D1
LCELVLDQGSCRDYSIRWYYDRTANACAQFWYGGCHGNRNRFDTEDECKKTCV
>P80163-D2
CSCSNWMDKECIYFCHLDIIW
>P80163-D1
CSCATFLDKECVYFCHLDIIW
>P0C8W6-D4
LAVEPAVEPAVEPAVEPTVEPAVELAVEPA
>P0CI21-D1
GSARGRCEAGNGPRLAGARTSRSRVVWECSPKRVVNSI
>P0CI21-D23
GSARGRCEAGNGPRLAGARTSRSRVVWECSPKRVVNSI
>P0CI21-D3
GSARGRCEAGNGPRLAGARTSRSRVVWECSPKRVVNSI
>P0CI21-D4
GSARGRCEAGNGPRLAGARTSRSRVVWECSPKRVVNSI
>B3EWP2-D1
PFFTVWNAPTKKCASQYGVDLDLSMFDIIHN
>Q2XXR8-D1
MRSSFCLFFFLLLVSYGSGAVITGSCTKDSQCGGSMCCAVSLWIRSLRMCVPMGMEGEECHPMSHKVPFGGKRLH
HTCPCLPNLACITTEEGNSRCL
>P0C6S3-D1
YCISGVIGHRRTECQ
>P0CH88-D4
IVGGNAASEGSWPWQVSL
>P0DMD6-D3
SGCFGHKMDRIGTISGMGC
>P0DMW6-D6
CTMPPVVGPCKGVFPRWYYDPTAGECKHFLYSGCKGNHNNFLQQADCANEC
>P22028-D8
VSSQPICGNRITEKGEECDVGLNAN
>Q90W97-D1
CQLAQDMGTCINFMLKWHYEASRKECSRFWYGGCGGNRNRFDTQEECEARC
>Q9TWF9-D4
CSLDMDMGRVCSSYESRWYYDRNAHECKHFWYGGCDGNGNRFMTKTECEETCG
>P86468-D16
CLNGGTCQDGYGTYKCTCPHGYTSTNCQ
>P0DP55-D1
SWTAIFTLFLCVLLYLSTVVDAYPPKPENPGENAAPEELAKYYTALRHYINLITRQRYGKRSTQEGILTDLLFGDSND
>P43318-D1
GFTYPGTLWCGAGNIADNYDQLGEF

>P0CI21-D10
GSARGRCEAGNGPRLAGARTSRSRVVWECSPKRVVNSI
>P0CI21-D25
GSARGRCEAGNGPRLAGARTSRSRVVWECSPKRVVNSI
>P0CI21-D9
GSARGRCEAGNGPRLAGARTSRSRVVWECSPKRVVNSI
>P0CH17-D1
LRKDTLCSSSFLSRSCCDSVCILI
>P86821-D1
ANTPEHAPEHAPEHAPEHAPEHAPE
>C0HJB4-D1
PCRSVEPMMRSGSCIRGW
>P0C8X6-D1
QCPSNCNKNGECVNGKCVCH
>P0CV87-D1
GCFGLKLDRIGSMSGLGC
>P22028-D10
MLGGPRCGNLYVEKGEECDCG
>P80163-D3
CSCINQLDSECYYFCHLDIVW
>P86468-D1
HHPCVNGGTCINSEPNEYNCACPEGYSGKNCE
>P86468-D6
CRNGGTCLAHSTKSYQCRCPEGFRGQWCE
>P0DMZ2-D1
FMSSYTDGMPWLRMTHHSPC
>P86468-D8
CKNGGTCQDGINDYICTCPPGYTGKNC
>P86468-D9
CLNGGRCIDRINNFHCLCPAGYTGRTCE
>P84038-D1
EGCEHGWKKFHGHCYKLFSRRHTWEDAEKDCRELSGHL
>P86468-D19
CLNGGLCMSPGVCICPPGYYGVSCEK
>P0DMB7-D2
PHVYSDDVTRCHVMSRENKKH
>P0DJ94-D1
HADGLFTSGYSKLLGQLSAKEYLESLL
>P0DMJ1-D1
VDICKIPKDEGTCAKFVLKWHFDSIEKSCKRFWYGGCGGNQNRFDTQKECEIACGKA
>P0CV91-D3
GLFIIDDKGILRQITINDLPVGRSIDETLRLVQAFQFTDK
>C0HJR6-D1
QERLPEYNITGRMMCSGYPEGGIDTC
>C0HJF4-D3
CALKKDEGPCKALKDRFYFDMEMFRCEPFEYGGCQGNENNFETI
>P0DN17-D3
CALKKDEGPCKALKDRFYFDMEMFRCEPFEYGGCQGNENNFETIEECEEMCLVKSK
>P86468-D4
CLNGGRCVDGIGHYTCVCPPGFTGERCE
>B5KL36-D5

CKGVPDSGPCFGMLHRYHYNSSIMTCQRFDYGGCMGNQNNFLTEKECLQTC
>P0DN12-D1
CRLPVDVGSCKMALQFWAFDSKIGKCVSFIYGGCDGNGNRFYTQKECEEYCGVSRD
>P0C6S2-D1
GQGRCIYKCMNHNLNSLQLHTLC
>A6MGY1-D1
CTLVLSQGTCRDYIIRWYYDKQANACAQFWYGGCEGNENRFDTEGDCKKTC
>P0DKR0-D9
LFEPTECGNGYVEVGEECDCG
>P0DMW7-D3
SAVCWAPARKGPCRAKLSRWYFVAEKGRCAPFTFGGCGGNRNNFESEEYCMAVCSS
>P85843-D1
HDPHTKHGRTSIVHLFEWRWQDIAAECERYLAPNGY
>P0DL27-D3
IVGGYECKPNSQPWQVSLNVGYHFCGGSLINQNWVVSAAHC
>P0DN42-D2
CMACGPRDSGRCFGPNICCAAGLGCSVGSPEALSCTEEDYIPIPCENGGRACGSKRGRCAAPGVCCNSGLCYFNTLVK
>P82972-D3
KSKNFSGCFGGRLDRIGSSSTLGCNAMK
>P83231-D3
SSSKKYSGCFGRRLDRIGSMSTLGCNTV
>Q2XXR8-D2
ACDRDVQCGIGMCCAVSLWLRGLRMCTPQGFEGDECHPFSHKVPFPGKRH
HTCPCLPHLVCTRYTDSRYRCTKDYKSI
>P0C8X6-D7
RSCPGNCNNKGRCVNGKCVC
>P86468-D3
NICLNGGTCKFDQKGQVNCLCPLGTSGLYCE
>P0DMH1-D1
CPANWVPFSGHCY
>P86468-D11
CLNGGTCVEGTDISYTCLCPKGFTGNNCEE
>Q9PS06-D1
CDHNWRKFHGHCYRYFTHRLNWEDAEKDC
>P0DKM8-D1
VCGTDGKTYTNECELRQTSCQEKKNIEVARPGSCDE
>P0DKM8-D2
VCGSDGLDYPSECALNMKACSTNKNIRLQHVGSCGE
>P0DKM9-D1
VCGTDGVTYADYCQLRTIACRQDKEITVKHLGQC
>P0DKM9-D2
CLDTCLQASDPVCGSDGQTYTSQCQMNAISCTLQKHIQIKHKGPC
>P86468-D12
CHNGGTCHNLVGGFSCSCPEGFTGMACER
>P00983-D1
ACHLPKAPGDCYGHYLRYYYDAAHGKCTTFAWTGCVGNGNRFLDLNHCNATC
>P86468-D17
CLHDGTCILDSSHSYHCACLAGYTGKRCE
>P0CH88-D3
IIGGIDATLGRWPWQVSL
>C0HJR6-D2

TNKMICAGLLQGGKDTC
>P0CH88-D2
IVGGQSASAGAWPWQVSL
>P86991-D1
FPDGGCGKRLPCAIDSN
>P0CV91-D4
GLFIIDPNGIIKHMSINDLPVGRSVEETLRLVRAFQFVE
>Q8T0W4-D5
CQAEPQVGLCRASIPRYYYTSGTCKRFRFGGCGGNSNNYNTEEECMKTC
>Q9TWF8-D4
CRFEKVVGHCRASFPRYYYDVTDQTCKTFVYGGCGGNNNNFKTKEECENAC
>P0CI21-D22
GSARGRCEAGNGPRLAGARTSRSRVVWECSPKRVVNSI
>Q2ES47-D3
PSICRLPVEKGLCFAISTRYFFNMASMQCQAFTYGGCGGNNNNFPDHISC
MEYC
>Q6T6S5-D1
DVCFLQVDEGPCLDDVPRFYYNTLTQSCEEFSYGGCEGNANNFKSYEACY
KTCFS
>E2E4L2-D1
LVPIAVVLCVLVCMAEAYPPKPEPPAGDAGPEELAKYHTALRHYINLITRQRYGKRSSPEVEMAELLFG
>P86468-D2
CFNGGTCEERFTGGYVCRCPPAYTGSNCEK
>P86468-D21
CLNGGRCIAPYECECPKGWTGKRC
>P0DMW7-D1
ALAVCSLESETGPCRASMPRWRFDIHLGKCVRFIYGGCAGNRNNFESEEDCMAVCKS
>P59068-D1
LWQLRGMILCVKPHSWPALDYADYGCYCGYGGSGTPLDDLDRCCQVHDKCYSDA
>P0DN43-D1
CYISNCPIGGKRAVQDLPTRQCMGCGPGDKGRCFGPNICCGEEIGCMVGTLEAMRCLEEDYLPSPCETGGKPCGSV
TGRCAAPGVCC
>P86468-D20
NPCLNGGVCARREGGYTCICRENYTG
>P0CI21-D19
GSARGRCEAGNGPRLAGARTSRSRVVWECSPKRVVNSI
>P0CI21-D20
GSARGRCEAGNGPRLAGARTSRSRVVWECSPKRVVNSI
>P0DM77-D1
CSQLKQAGTMCRSAAGSCDLPEYCTGGSPYCPSNVY
>P0CI21-D13
GSARGRCEAGNGPRLAGARTSRSRVVWECSPKRVVNSI
>P0CI21-D14
GSARGRCEAGNGPRLAGARTSRSRVVWECSPKRVVNSI
>P0CI21-D16
GSARGRCEAGNGPRLAGARTSRSRVVWECSPKRVVNSI
>P0CI21-D17
GSARGRCEAGNGPRLAGARTSRSRVVWECSPKRVVNSI
>Q6T6T5-D1
LCNLPAVQGPCRNWEPRWAYNAVTRLCQAFVYGGCRGNSNNFRSKAECQASCPRQSSR
>P13211-D4

CSCENLKDRECVYFCHIGIVW
>P86468-D15
CMNGGTCQDSYGTYKCTCPHGYHGFNCQ
>P0DN20-D1
CNLKMEPGSCFARQAMYYYDSQEKACRMFLYGGCQGNGNRFDTKEDCEKMC
>Q90WA0-D3
CVDPPVTGPCRASMPHWFYDPLKQTCFRFTYGGCAGNANRFGKKDDCMNVCA

Data S2. The medium-length toxin proteins in the yellow catfish.

>Za2G-D17
TAVTPGINFPEFTIVGQVDGGHIEYYDSNIMKPIPKAEWKKKVTADDPDYWNKQTQSTQETLKDNVIILKVNTHTHT
QGCVLHRGVHTMQVMYGCELDDDGTVRGYTQFGYDGEDFLSLDLKPQAVISKNKWDNDRGAIVGEKNYLENTCI
EWLKKYVSYGKETLERKGK
>Za2G-D3
MSLCSTVMKTLIFFTFSLHLSSAVTHSLQYFYTGVTPGINFPEFTNVGLLDGQQISYYDSNIRKKIPKTEWIQKMTADD
DYWNSGTQNLQDTQESFKVNVATAMQRFNQTTGVHSVQLMYGCELDDDGTVRGYRQYGYDGEDFISLDLKTLTW
TAAKPQAVITKNKWDHNPGVSVGRKNYLENICIDWLKKYVSYGRETLERNGK
>Za2G-D22
VTHSLQYLYTAITPQINFPEFSAVGLVDGGQFVYYDSNIRKMIPKTEWIRSDKISAYDSDYWKRETERSRSDHEDLHHL
LHTVMKSFNHTQGVHTFQRMYGCELDDDGTVGGYNQFGYDGEDFISLDLKTGSWTAAKPQAVIIKNQWDSTGAQ
GQYWKSYLETECIEWVKKLVSYGRETLERKVRPEMSLFQK
>Za2G-D38
TYNKVLIFLTYCVHLSLADTHALQYLYTALTSGINVPEFIAVVLVDEEQSVYYDSNIRKMIPKTEWIQKFSADDPKYW
NRETERMQNDQEDYKVDKTTLMHRFNQTTGVHTLQRMCGCELDSGTTGGHNQFGYDGVDFISLDLNTETWTVN
NNKAEIFIKEWDPEGEKAKYWTTQLTYECIDQLKKFVSYGRETLER
>Za2G-D40
TILKVLPFLTFTLHVSSADTHSLQFFYTAGTPGIKVTAVGLLDGEQFVYYDSNIRKMIPKTEWIQKISTDDKDYWNRET
EHVEDNQDSVATVMKNLNQAEGDHTLQWMFGCGLDNGTIRGYSQYRYDGEDFISLDLNWCQKPGCRTWTAANE
KAEIFIKKWDPRRDQARYWMDYLQFNCIDQLKKFVPHSRETLERKDPPTASVIQK
>Za2G-D44
IPKTEWIKKVDADNPYWNSTETEVWQDEEEWLKHDVVTAMNHFNQTEGVHTVQLMFGCEIDDDGTTRVFSHFGY
DGEDFISLDLKTKTWTAASIKTLITKKKWNPTGHKAKGCTLYLENECIDWLKKFVSYRRDTLERKVCPTASVFQK
>Adamalysin-D5
FSQKYIERFYSFQKGPPGHKKRSHPSFSSKLKDMQSFFGLDQTGNLDTETLDEMKRPRCGVPDIEEYVYNRGNRWKK
NVITYNIGKYTSDLPVSTVDSLIGAALDVWANASPLRFFRSSSQQADIMVEFGSKYHGDNYPFDGPKGTLAHAFDPG
EGVGGDVHFDDDELWTADSRGFSLYLVAAHEFGHSLGLKHSQNPQSLMYPTYRKRQPQNMLSSEDILNINALYGIN
>Za2G-D43
FFHIFVQLSSADTHSLQFLYTAVTPGINVTAVGLLNGEQFVYYDSNIRKMIPKTEWIQKISTDDKEMTGTEKPSVVKD
HQYQLQDIVTTVTKRLNQAEGDHTLQWMFGCGLDNGTIRGYSQYRYDGEDFMSLDLNWNQEHGTWIAANEKAE
LFIKEWNYKEHAIYWMNYLKTECIDRLKKFVPHGRETLESKGK
>Za2G-D27
SVTHSLQYFYTAVTPGINFPEFTAVGLVDGGQFVYYDSNIRRMIPKTEWIQKMNYDDPDYWNRETQNLQGSEETFKV
NVDILMMRFNQTTGVHSVQVMYGCELDDDGTVRGYRHYGFDGEDFISFDLKTVTWIAPKPQAVITKNKWDNYPG
MTVARKNYLDDICIEWLKKYVSYGKETLERKVRPEASVFQ
>Za2G-D31
VTHSLQYLYTAITPQINLPEFTAVGLVDGGQIVYYDSNIRKMIPKTEWIQKISADDPDYWKRETEHSRSDHEDLHHLL
HTVMKSFNHTQGVYTLQRMYGCELDDDGTVGGYNQFGYDGEDFISLDLKTGRWTAAKPQAVIIKNQWESTGAQG
QYWKSYLETECIEWVKKLVSYGRETLERK
>Za2G-D32

MDLSCSLVKVLLFLMNGFHQTSAVTHSLQYFYTAVTPGINFPEFTAVGLVDGGQFVYYDSNIRRMIPKTEWIQKMNY
DDPDYWNRETQNLEGSQETFKVIIKIIAVYTNYVYTAMQRFNQTTGVHSVQMYGCELDDDGTVRGYRQYGYDGED
FISFDLKTVTWIAAKPQAVITKNKWDNDPGMSVARKNYLENICIEWLKKYVSYGR
>Za2G-D46
MSPRSTVMKTLIFFTFSLHLSSAVTHSLQYFYTAVTPGINFPEFTAVGQVDGQQFGCYDSERREVIKTEWIHSDHPDH
WNRITRIAKEHQEAYKAHVFTQMQVFNQTTGVHTVQRMVGCELDDDGTVRGYRQYGYDGEDFLSFDPKTLTWTA
AKPQAVITKNRRNTEPGNNNEMKNFLEIDCIDWLKQYVSYGKETLERKVRPTVSMFQK
>Za2G-D5
SNHLKSALYVSVTHSLQYFYTAVTPGINFPEFTAVGLVDGGQFVYYDSNIRRMIPKTEWIQKMNYDDPDYWNRETQ
NLLGSEETFKVNVYTAMQRFNQTTGVHSVQLMYGCELDDDGTVRGYRQYGYDGEDFISLDLKTVTWIAPKPQAVIT
KNKWDNDPGMTVARKNYLENICIEWLKKYVSYGKETLERKVRPETSLFQE
>Za2G-D8
SVTHSLQYFYTAVTTGINFSEFTAVGLVDGGQFVYYDSNIRRMIPKTEWIQKMNYDDPDYWNRETQNLQGSQETFK
VNVYTAMQHFNQTTGVHSVQVMYGCELDDDGTIRGYRQYGYDGEDFISLDLKTLTWIAPKPQAVITKNKWDNDP
GMTVARKNYLENICIEWLKKYVSYGKETLERK
>Za2G-D9
MSLCTVMKTLIFFTFSLHLSSAVTHSMQYFITAVTPGTNFPEFTIVGLVDGGQFVYYDSNIMKMIPKTEWIDVMGEDY
WNREAQKQQQSQETFKADVSTVMQRFNQTTGVHTVQVIYGCELDDDGTVRGYRQDGYDGEDFISFDLKTLTWTA
AKPQAVITKNKWDNDSGYNVARKNYLENECIEWLKQYMSYGRETLERKVRPTASVFQK
>Za2G-D2
MSLCSTVMKTLIFFTFSLHLSSAVTHSLQYSYTAVTPGINFPEFAEVGQVDGGQISYYDSNIRKKIPKTEWIQKVTADDP
DYWNSGTQILQGAQDTFKVSVSSLMQRFNQTTGVHSVQVMYGCELDDDGTVRGYEQYGYDGEDFISLDLKTVTWT
AVKPQAVITKNKWDNDHGATVGEKNYLEKDCNEWLKKYVSYGRETLERKGK
>Za2G-D47
MSLCSTVMKTLIFFTFSLHLSSAVTHSLQYFYTAVTPGLNFPEFAEVGQVDGGQISYYDSNIRKKIPKTEWIQKVTADD
PDYWNSGTQNLQGAQETFKVSVASLMQRFNQNNGVHSVQVMYGCELDDDGTVRGYEQYGYDGEDFISLDLKTVT
WTAAKRQAVITKNKWDNIPGYNMDRKNYLEKECIEWKYVSYGRETLERKVRPTASLFQE
>Adamalysin-D3
MKTYYQLCILVALVFRVRSNPVPQNNDLTDEEFAKNYLKRLYNMKEVNKPSFGRTTSEMSLKLSQMQQFFGLKVTG
ILDDETIAMMKKPRCGVPDVAAFKSNALPIKWSTNSLTYRIENYTPDMSVAEVDETIERALQVWARVTPLRFTRINSG
VADIMISFGRGSHGDAYPFDGPSGTLAHAFAPSSGIGGDAHFDEDENFTFSSTNGFILFLVAAHEFGHSMGLSHSSDP
GALMYPTYSYRDPKTFVLPADDVKGIQSLYGPNPDKPVDPSKPNPPPV
>Za2G-D41
TLIYLLLWNSFSVHLSYAVTHTLQYIHTAVTPGINLPEYTDVGLVDGEPFVYYDSNIRKYIPRTEWINKITDYDPDYWS
SGTQNQNYVQEIYKDDVITLMRRFNQTEGVHILQRIYGCELHDDGTVRGFEQLAYDGEDFMSLDLEHVTWTAVKP
QALNTKNKLNTEGAAHFEKFYLENICIEWLQKYMTYGRDTLERKGR
>Za2G-D48
IIFFFFTVTVTHTLQYIRTAVTPGLNLPEYTDVGLVDGEPFVYYDSNIKKYIPKTEWINKITDDDPDYWSSGTQNQNYE
QEIYKENVITLKRRFNHTEGLHTLQRIYGCELHDDGTVRGYEQLAYDGEDFMSLDLERVTWTAVKPQALNTKNKLN
TEGAATFEKFYLENICIEWLQKYVIYGRETLERKARPEVSVFHK
>Za2G-D1
MSLCSTVMKTLIFFTFSLHLSSAVTHSLQYFYTAVTPGINFPEFTDVGKVDGQQFSYYDSNIRKKIPKTEWIQKVTADD
PDYWNSGTQNLQGQQEIFKVNVASLMQRFNQTTGVHTVQRMYGCELDDDGTVRGYTQYGYDGEDFLIFDLKTVT
YTAPKPQAVISKNKWDNDRGDTVGQKNYLENTCIEWLKKYVSYGRETLERKDRPTASVFRK
>Za2G-D6
TLCSTVMKTLIFFTFSLHLSSAVTHSLQNFYTGVTPGINFPEFTAVGQVDGGQFVYYDSNIRRETPKTEWIQKVTADDP
DYWNRETQILQGNQENFKANVATLMQRFNQTTGVHTVQVMYGCELDDDGTVRGYTQYGYDGEDFLIFDLKTSTW
IAPKPQAVITKNKLDNNPGVSVGRKNYLENICIEWLKKYVSYGRETLERKDRPTASVFRK
>Za2G-D18

VTHTLQYIHTAITPGINLPEYTDVGLVDGEPFVYYDSKIRKYIPKTEWIKRITDDDPDYWSSGTQIQNNAQEIYKVNVI
TLMRRFNQTEGVHTLQRIYGCELHDDGTVRGYEQLAYDGEDFISLDLEHVTWTAVKPQALITKNKLNTEGAAIFEK
VYLKNMCIEWLQKYVIYGRETLERKDRPEVSVFHK
>Za2G-D25
SVTVTHTLQYIHTAVTPGINLPEYIDVGLVDGEPFVYYDSNIKKYIPKTEWIKKITDYDPDYWSSGTQNQNDEQEIYKD
DVITLMRRFNQTEGVHTLQRIYGCELHDDGTVRGYEQLAYDGEDFISLDLEHVTWTAAKPQALITKNKLNTAGAAT
FEKLYLENICIEWLQKYVSYGRETLERKARPEVSVFHK
>Za2G-D49
VTHSLQYFYTGITPGIHFPEFTAVGLLDGQHFGCYDSKRREIIKTEWISFDDPDHWNRITGANDHQVAFKAHVITQM
QRFNQTTGVHTVQQMYGCELDDDGTVRGYRQYGYDGEDFISLDLKTLTWTAAKPQAVITKNKWDNNPGATVGEK
SYLEDTCIDWLKKYVSYGRETLERKVRPETSLFQK
>P28891-D3
SLSQMYIALNIRVVLVGLEIWSVVNFINIDGSAGEVLGRFTQWREKELVHRRRHDSAQLILNKGYGSTAGMAFVGTA
CSRSHGGGINAVLSTFASIVAHELGHNLGMNHDDDGRVCRCDTGNCIMNSGATGSRNFSSCSADDFEKLILNTGGT
CLLNVP
>P31989-D2
VAATMAHEMGHNFGMSHDSEGCCQALPKDGGCIMAAATGYPFPSVFNQCNQAELKRYLNSGGGKCLFNLPNTR
VMYGGQRCGNGYLEEGEECDCGEVEECSSPCCNANNCTLKAGAECAEGVCCENCR
>Q1PHZ4-D1
VFQFYRVLNIRVALVGLEVWSDSDKCAVTQDPFTTLHEFLDWRKLKLLPLRPHDNAQLISGVYFQGTTIGMAPIMSM
CTAEQSGGIVMDHSDNPLGAAVTLAHELGHNFGMNHDTPERGCGCGMTVERGGCIMTPSTGYPFPTVFSTCSKKD
LAASLDKGVGMCLYNIPEVKVLYGGQKCGNGYVEEGEECDCGEPEECVNPCCNATTCTLKEDAVCAHGQCCEDC
KLKPAGTPCRELSNSCDLPEFCTGSNPHCPANVYLHDGHACHTVDGYCYNGICQTHEQQCITLWG
>B5U6Y3-D1
SLQYNSQHYCGGTLIHPQWVVSAAHCWKPNYLIKVVLNEYDLFKKEGVEQVFNVSKTLVYYLYNYRTFDNDIMLL
KLEQPADLNSNVQPVKMPSTDTPPTFGGMLCVVSGWGVTQVYSYYLSPMLRAVDVQIIPNCQNYYLYRITDNMVC
AGSPLGGKDSCQGDSGGPLVCNGYLEGIVSWGIGCASPYFPGVYTKVRNYIRWINLTIDAN
>T1DKS4-D1
MIIFKDIISGDEMFSDIYKLKESENGMMIEVEGKMISRSEGDIDDSLIGGNASAEVQDDGCDSTTVSGVDIVLNHKLQE
TSYDKKSYMVYIKDYMKAVKTKLETCPDRVEPFMANAPAEVKKIIGNIKNFQFFTGESMNPDGSIGLLDFREDGVTP
YMLFFKDGLESEKC
>P30894-D4
RGELSVCDSISQWVTAVDKKTAIDMSGQTVTVLEKVPVANGQLKQYFYETKCNPLGYTKEGCRGIDKRHYNSQCRT
TQSYVRALTMDSKRKIGWRFIRIDTSCVCTLTIK
>Q91516-D1
DENGFCGGTLINQRWVVTAAHCLQETPDHVTLGDFDKFRPDAGEQKIKVEKVVVHPHFHEYTFDSDVALLYLAEP
VVFSSVVSPVCLPNTHLAKRLERPGENGLVSGWGATHFLGRSSRFLMKVSLPVVDQKECMDSTDQVITDNMFCAGF
LRAEKDACSGDSGGPFVVNYRGTWFLTGVVSWGEQCAADGKYGVYTRIRNFLHWIE
>Q9I8W9-D2
CGGSLINEIWVLSAAHCFQSSSTSGITINLGMESLELTNSNQQQRSASSIIINQNYDSTTKDNDIALVQLSSSVTFNNYIQ
PVCLAASSSSFPAGTEVWVTGWGTIASGVSLPSPQTLQEVQLPIVSNSDCTTSYGNGSITGNMMCAGLAQGGKDSCQ
GDSGGPLVVQENGVWVQAGIVSFGYGCALPNIPGVYTRVSQYQDWISSWI
>E0Y421-D1
CGGILIHQQWVVSAAHCWRPSNIIQVVLSAHNLVAEDGSEQVFNVSRIITNPTYNLKTYNGDIMLLKLSQPAVLNAY
VQPAPLPDDSTPPLDAGTTCTVSGWGVTRVYSFFLSPVLRAVDVDYIPNCYYFYYFRVNENMICAGSRYGGKDSCQG
DSGGPLMCNGILEGVVSWGIGCANPYYPGVYTKVRNYSKWISGIISSDS
>Q8JH85-D6
CGGTLINKYWVLTAAHCNVGVGNMRIVAGDYSVSVYEGNEQYRSPKLLVPHPLYNKTTNNADIMLIKMQTPVTVN
QFVFPAPLPRQGDFMPEGRVCRVSGWGFTSPTGGIPTSLHTATVPIVSSTHCNSTDSYNGNITENMICAGYAEGGTDA
CRFITLPSLQGDSGGPLVCEGRVYGIVSWGNSCADPKYPGVYTAVSKFRTWIDSTVFGN
>Q3HXY9-D1

VDPKLFNKRRYRSPRVLFSELPPDSEPTEHQGSKDRTKRRAGQPQSRGVYSVCESVSFWVGNKTKATDISGNEVTVLP
DVNINDVKKKQYFFETVCSGARTGGSGCLGIDVRHWNSYCTDSHTFVRALTSFKNLVAWRLIRINVACVCVLSRKS
>Q8AY75-D1
PEQITEYKGVFEMFDEEGNGDVKTQELERLMSLMGINPTKRELSQMAKNVDKDGKGTFNCDRFLGLMALYHERAK
NQDAELRAAFKVFDKEAKGYIDWNTLKYVLMNAGEALSEEEAEQMMKEADKDGDGTIDYEEFVAMMTGDSFKM
T
>P85031-D2
VQVYCDMTTDGGGWIVFQRRQNGLTDFSRKWSEYRAGFGNLEDEFWLAQGRYELRIDMRNGQEAVYANYDRFSI
GDSRSLYKLRIGEYNGTAGDSLSYHHGRPFSTKDKDNDNAVTNCALSYKGAWWYKNCHRVNLNGKY
>P82942-D1
YKPLNTFIALIGLEVWTDSDKIAVTTPSSDTLGAFTKWRNEDLVKRIKHDNAHLITGIDFEGSTVGLAFIGTLCTGHST
GVIQNHNPNAIAVGATLAHELGHNLGMNHDTSSCVCTEDSCIMTAALSYTIPRLFSSCSINQFEQYLNSRSPECLFNK
PQPTTLILPPVCGNGFQESGEECDCGSVKIADATLMCRAKRDECDLPEYCTGNSPSCPEDVFAVNGLQCKNGDGYC
YNGQCPRLQDQCIKMWGPNAVVGDDFCYNQNTRGTYYAYCVWPINGPYIACQKQDVKCGKLFC
>D8VNT0-D2
RDCSDIYASGQREDGIYSVFPIHFPSGFQVYCDMSTDGGGWTVFQRREDGSVNFYRDWAAYREGFGKITGEHWLGL
KRIHALTIQANYELRIDLEDFENSTSFAQYGSFGVGLFSVDPDEDGYPLSIADYSGTAGDSLLKHNGMKFTTKDKDND
HSENNCASFYHGAWWYRNCHMSNLNGQYLRGQHSSYADGIEWSSWTGWQYSLKFTEMKIRP
>P30894-D3
RGEFSVCDSMSHWVTDKTTAVDIHGYEVSVLTEVEIKRSTMKQYFYETTCQNSKPIKSGCRGIDDKHWNSQCKTSQT
YVRALTKYNNVMNWRWIRINTSCVCALSRKH
>P67860-D1
VVPFTEVYNKSMCRPREMLVEVHQEYPEDIEHIFIPSCVVLMRCAGCCNDEMLECKPTATRNITMEVQKLKPMRIKR
NYPMSFAEHTECECRVKKAMQENTDKKPRKGKGKGQKGKRKKNRDKMRDFIHCKPCCSTCSERKKRLYVQDPET
CQCTCKHSEADCKQKQLELNERTCRCDKPRR
>F8S116-D2
FCGGTLIDAQWVLTAAHCLERPSAYKVYMGIHTERATEASKQIRDLDQIIKGPPGTDIALLKLDRPANLNDKVAKVC
LPQKDYIVPSGTECYVTGWGETQGTGGEGILKETGFPVIENKVCNRPEYLNNRVKDFEMCAGNIEGGTDSCQGDSG
GPLVCYGQNTFILQGVTSWGLGCANAMKPGVYARVSKFTDWIE
>Q6T269-D1
QWCALKKDEGPCKALKDRFYFDMEMFRCEPFEYGGCQGNENNFETIEECEEMCLVKRDEPGPCRAVVPRYFFDSKV
NECRRFFYGGCFGNANNFKTLKECKDRC
>Q90W38-D2
VDPKLFNKRRYLSPRVHFSDVVPFDGHTEPRSPRTRRKVRDFQNRGEYSACDSENHWVGNMTRATDLAGNEVTVL
PDVRINNVVKKQMFYETTCRVTNRGSTQRGMKAGTTGCRGIDNKRWNSYCTNTHTYVRALTSFKNQVTWRFIRIN
AACVCVVSRKS
>P85031-D3
PLQVFCDMTTDGGGWIVFVRRQSGKVDFFRNWRNYTAGFGDMNDEFWLVDLRDKGEHAYAQYDKFSVSEPRSRY
KVHVGGYSGTAGDSMTYHNGRPFSTYDHDNDIAVTNCALSYKGAFWYKNCHRVNIMGRY
>Q8AY81-D3
CGGFLINPSYVLTAAHCKSRHYHNFLNVVLGSHNINPERNDLKRYTVEKVHVHPFYKIKPDLGYDIMLLKVSKEISP
NDVHVKTIEISSKHPDNNINCQVAGWGKTEDQVLSPDLLETDVTIINITVCEKEWTKADLFKLPDNILCAGGYETKS
GACQADSGGPLVCNGVAVGIVSFNNNSNCKYPELPNVYTDISAYIDWINSVI
>P0DP54-D2
LELSGVVKCSTGRSTLAYIMYGCYCGVGGEGWPRDPADWCCHKHDCCYAKAEDQGCYTKTHTYPWSCDSQSLEC
GSLTDRCEKMLCVCDREAAKCLKKAPYNLKYVAWPDFLCGPELPTCAYY
>P0DP54-D1
IIHSLLVSMASLVDQRNVRSKRGLLELASIIKCTTGRSAFSYLMYGCYCGLGGKGWPRDRADWCCHKHDCCYGDAE
VAGCHTTTDKYQWTCEDKEADCDSLKDRCEKILCRCDREAGRCLRKAPYNRKYAYWPDFLCGCLYPTCNIY
>Q072L7-D6

VYNGHFCGGSLINKDWVLTAAHCFSSMSSLTVYLGKQTLKGSNPNQIARSVKQMIIHPNYNSATHDNDIALLLLSSS
VTFTNYIRPVCLAGQGSSFPAGTNCWITGWGSIASGVQLPSPGVLQEAVVPTVNSFICDYLLGYGSITTNMICAGYLQ
GGTDTCQGDSGGPMVAKKGAVWIQTGITSWGEGCARAFSPGVYTLVSQFQTWISSVINQN
>D8VNT0-D1
RDCSDIHASGQRENGVYSVFPTHYPAGFQVYCDMSTDGGGWTVIQRREDGSVNFFRDWDSYREGFGKITGEHWLG
LRQIHALSIQANYELRIDLEDFENSTAFAQYDMFGVGLFSVDPEDDGYPLTIGDYTGTAGDSLLKHNGMKFTTKDRD
NDHSENNCASFYHGAWWYNSCHMSNLNGQYLHGQHTSYADGIEWSSWTGWQYSLKFSEMKIRPTREE
>T1DKS4-D5
VSGIDIVLNHKLQETSYDKKFYMVYIKDYMKAVKAKLQETCPERVEPFVAIAPAEVKKTIGNIKNFQFFTGDSMNLD
GSIGLLDFCEDGLTPYMLFFKDSLELGKC
>C9E1S1-D2
TLLQFYRALNIRVALVGLEVWSDSDKCPVSQDPFTTLHEFLDWRKLKLLPQRPHDNAQLISGVYFQGTTIGMAPIMS
MCTAEQSGGIVMDHSDNPLGAAVTLAHELGHNFGMNHDTPERGCGCRVTAERGGCIMTPSTGYPFPTVFSSCSKK
DLLVSLDKGVGMCLFNMPEMKVLYGGQKCGNGYVEEGEECDCGDLEECMNPCCNASTCTLKLNAVCAHGQCCE
DCQLKTAGTLCREPANSCDLPEFCTGSDPHCPANVY
>T1DKS4-D4
GGGVSAEVQDDGCDLMTVSGIDIVLNHKLQETSYDKKFYMVYIKDYMKAVKAKLQETCPERVEPFVAIAPAEVKKT
IGNIKNFQYFTGDSMNLDGSIGLLDFCEDGLTPYMLFFKDSLELGKC
>C0K3N5-D2
VVPFMEVYTKSRCNPRETLVDVQHEYPHDTHVTYLPSCVVLQRCGGCCNDEALECVPTHTNNVTLELYRVKPGVG
EHKTLLSFTEHTHCDCRVKPEVKTKKEYRCEPCSERKKHWFVQDPLTCRCSCTLTQLQCRSRKLELNERVCR
>T1DKS4-D3
EGDINDSLICGNVSAEVQDDGCDLMTVSGIDIVLNHKLQETSYDKKFYMVYIKDYMKAVKAKLQETCPERVEPFVAI
APAEVKKTIGNIKNFQYFTGDSMNLDGSIGLLDFCEDGLTPYMLFFKDSLELGKC
>T1DKS4-D2
EGDINDSLIDGNVSAEVQDDGCDLMTVSGIDIVLNHKVQETSYDKKFYMVYINDYMKAVKAKLQETCPERVEPFVA
IAPAEVKKTIGNIKNFQFFTGDSMNLDGSIGLLDFCEDGLTPYMLFFKDSLELGKC
>E0Y419-D1
CGGSLINANWVLSAAHCFQSSSTSGITIKLWKVWSLANSNQQQRSASSIIINQNYDSTTKDNDIALVQLSSSVTFNNYI
QPVCLAASSSSFPAGTEVWVTGWGTIASGVSLPSPQTLQEVQVPIVSNSDCATKYSSITDNMLCAGLAQGGKDSCQG
DSGGPLVVKRNGVWVQAGIVSFGHGCALPNIPGVYTRVSQYQDWINSNIGSN
>Q8UUJ2-D1
FCGGSLINERWVLTAAHCFPSFSLLGITANLGLSNLLGSNSNKQQRAIAALTIHPRYNSINNDNDIALLQLTSPVTFTN
YIRPTCLAASTSDFPHRTNVWVTGWGDIKSNVELPFPQTLQEVKVPIVSNSDCAKSYGNDIITYNMMCAGLSEGGKD
SCQARNQHYHIISGHSTGLHGRKADIFGYGCALPNFPGVYTRVSQYQDWINSII
>P16354-D1
AWMIPGTLWCGSGNKASDFSDLGLFEDTDKCCREHDHCEQTISSFQFGYGVFNSHFFTLSHCNCDSKFRRCLHNAN
DRMSDMVGYGYFNVLKMRCFEFSQRLECAERTWW
>P80966-D1
QPRALWQFGKMITCVQPNVNPFIYNNYGCYCGFSGSGSPKDQIDQCCLIHDKCYENARKHPDCPGVANLPYVKVY
NFSCSDKTITCSASNDKCQAKVCECDQVAANCFFQHNHTYNPNNKNL

Data S3. The long-length toxin proteins in the yellow catfish.

>A7ISW1-D1
HTANTLSSDDLINVLAQTDIDRMWKNDLKPMLVVRYPGSPGSQAVQQRIKSTLSSLNAGWEVTEDRFQSWTPYGQ
MPFNNIIATLHPGSKRRIVLACHYDSKYFPPQWHGREFLGATDSAVPCSMLLEMARALDNELKTLKVRSCGSDLTLQ
LIFFDGEEALYQWTSTDSLYGSRHLAAKMENTAHPVGATDTNQLDGIDLFVLLDLIGGPMPHFGNQFSNTARWLSK
LQNIERRLHTLGQLEDHPNEVQYFWPGMPVGPVQDDHMPFLSKGVRVLHLIPTPFPSVWHTFDDNEENLDRATIQ
NLNKILQVFVFEYLNM
>F8S0Z7-D1

PLLLLLCASCWLAAADWELTILHTNDVHARVEETNKDSGKCTKGGCYAGVARRFTKIQEIRSQKKPLLLLDAGDQF
QGTVWFNYYKGSEAAHFMNRLGYDAMALGNHEFDNGVDGLKPFLQAVNCTVLSANIKAVEPVASQISGYYSAST
VLKVGTESVGIVGYTSRETPSLSMPGPYMQFEEEVAALQLEVDKLITTSGVNKIIALGHSGFETDKEIAKRVRGVDVVV
GGHSNTFLYTGDHPSSEVPAGPYPFMVQSDDGRQVPVVQAYAFGKYLGYLKVTFDSDGKVVKADGNPILLNSNIAE
DPSIKAEVDAWKGKLANYSAQFVGKTLVYLNGTFEECRFRECNLGNLICDAMVHHNIKYPDELQWNHVSACILNG
GGIRGPIDERSRNGSITMEDVLSVLPFGGTFDLVMLKGSTLLQAFEHSVRRYGGNTGEFLQVSGFQIEYDLSKPSGERV
KKVSVLCTECRVPHYEPLDTKKAYRVVMPSYLVDGGDGFSMIKEEKLKHDSGDLDMSVFAGYITERQRVHPSVEGRI
>F8S101-D1
AAVHKATVYWDPDHKTVLLKDGVLDDTGDAYGFYNDSLSETGWGVLEIRAGYGRTPRPDDKTFFLAGYLEGFLTA
RQMVDHYTNMYPQLITKPNILQAVKTFIIKQSDWSRQQVKLNKTSDPLWAHTGFILAQLDGLQAGAAEWAKRSGG
KALSRFEVHFLNAVGDLLDLIQMLVPEAKPSLRGYKQPPMGHCSALIKMLPGYENMLFAHSSWYVYAATMRIFKH
WDFNVQEPHTATGKVSFSSYPGFLVSLDDFYLLGSGLMMTQTTNNIFNQSLYESVTPFSLFAWQRVRLANALAHTGE
EWAKIFSRFNSGTYNNQYMVVDVSKVSLGSRLDDGALTIVEQIPGLVEYSDQTEALRFGYWPSYNVPFHQKIYTLSGY
EEMWMDYGEDFSYELCPRAKIFRRDQASVTDLSSLKHIMRSNDYKKDPYSKGDACKSICCRGDLRTSNPTPEGCYDT
KVTDLRMAQQFRSEALNGPSTEGDLPPFTWDAFNSTMHQGLPRFYNYTFITMHPVL
>J3RZ81-D1
DQQVKYNMRKSEDLDDEGCFLQKGHNEVLQQCGFNTTAKTILIIHGWTIGGVFESWMYKLVSAVVQREAEANVIV
VDWLGLAHQLYPDAVNHTLRVGHSIATLLDWLKDEQQLPLENVHLIGYSLGAHVAGYAGTFVNGKVGRITGLDP
AGPMFEGAEPNKRLSPDDADFVDVLHTYTREALGVSIGIQQPIGHIDIYPNGGDVQPGCALGDVLSSAAAGDFMEV
MKCEHERAVHLFVDSLMDKEHMSFAYQCTGPERFNKGICLSCRKNRCNNVGYNTRSVRRTRNSKMYLKTRADTPF
AGYHYQMKMHVFNRKHSDDADPTFYIKLFGAHNDTNDIYVDIADGVGLNLTNTFLIFTEEDIGELLKIRLTWESPTE
SFSAVWKHIKSFWSTSSSTKVLQVRRIRIKCGESQRK
>J3RZ81-D2
DINDACTVKLFQAHTLQSCSFNSSHPLVIIIHGWSMDGMIESWVTRLAAALKSTQKDINILVSDWMTLAQQHYPIAV
QNTRVIGQEITQLLMWLEDLTQFPVSKAHLIGYSLGAHIAGFAGRNLATSGRTLGRITGLDPAGPLFEGMSSTDRLSP
DDARFVDAIHTFTQQHLGLSVGIKQPVAHYDFYPNGGSFQPGCHLQVKNLYTHLSQYGLMGFEQTMKCAHERAV
HLFIDSLLNRDKQIMAYKCRDSAAFNKGICLDCRKNRCNTLGYGINKVHTSTSKRLYLKTRSLMPYKMYHFQFRIQL
FTQFENTDLSLTIKLTGTLEESEALPITLVKVSGNKTYSFLMTVDTDIGDLMMVHVSWEAESVWTNMWSKMKTILPW
GSKEDEPQLTIGKIRVKAGETQQR
>J3SDX8-D1
SEIIRHWGYPAEEFEVVTEDGYILSINRIPHGIQSKFQEEPKPVVFLQHGLLAAGSNWVTNLPNTSLGFLLADAGFDV
WIGNSRGNTWSRKHVRFNPKQKEYWQFSYDEMAKKDLPAVINFITKTTGQEQIFYVGHSQGTTIAFMAFSTMPELA
SKIKMFFALAPVATVAFTESPMGKLSILPEFVIWKLFGNKDFLPQSALIKFFATQFCSKRPISILCGNIFFLLCGFDERNL
NMTRTPVYTTHCPAGTSVQNMVHWSQAIKSKKLMAYDYGKAGNIVHYNQSTPPLYNVQDMKVPTALWSGGHDT
LADPKDMAVLLTQIPNLVFHRNIQHWEHLDFIWGLDAPQEMYEPMIKLM
>P81428-D1
VFIRRKVAHGVLSRAKRANSGWFEELKMGDLERECIEEKCSYEEAREVFEHTEATSEFWNVYHDGDACISQPCLNKG
VCKDGLGRYSCYCPDQFQGYNCEIAIQQLCIVNNGGCEHFCTVEKKTGTVFCSCADGYKLADDRKACTSDMITSAN
MTITIDTQSQAKSNSLFGLMDPSPTDKQIPELPESDGEKRIVNGEDCPPGECPWQALLINEDKIGFCGGTILNEYFILSA
AHCMKLSRSITVILGETYTRSWEGREAVHEVEQVLVHANYKPDTFHNDIALIKLVKPIQFSKYIIPACIPDHDFAENVL
MQQDEGLVSGFGRLQEGGIQSTTLQRLSVPYVDRSVCKESTKYNISPRMFCAGYGREEKDACQGDSGGPHVTKYKN
TWFVTGVVSWGEGCARKDKYGIYTQVSKYLKWIEGVM
>Q90YA8-D2
SHKPRKLPLAQVRNLAAQVDITRLWETHLRPILIERVPGTAGSKAVLQHIVSQLRSLSAGWTVEEDSFQSSTPKGPVTF
SNVLAVLDPSAPRRLLFACHHDSKILPRDPKDPQRVFIGASDSAVPCAMLLELASALDTELKSLKQQRSALTLQLVFF
DGEEAFEEWTDTDSLYGSRHLAELMAHKPHPPGSTHTTQLQAVDLFVLLDLLGAAEPLIVNHFDNTAHWFDRLIA
AERRLQRQGLLSSQGSEKNYFRKDVYLGPVQDDHVPFLLRGEVPVLHLISTPFPPFWHTLEDTEENMHRPTVENLTKI
LAIFLAEYLHL
>Q92031-D1
KNPSAVEYALDIDGKTFTISLEKNRELLAKNYTLTYYTEDGAKEITYPSNVDHCYYHGQIQNINDSSVSVGMCSGMR
GFLRAENQVYLIEPLEDSVKGDHAVYKQEHLRTKRATHGYINDTVYDYGVAPRLAGLQKSISSCYITCAWICNKTKN

VLGNVLLNMLFNNIYFQLYRPLNIRVMLVGLEVWSNKDQIDVSNVPDHTLDRFLKWRQTDLLPRKKHDNAQFVTG
MDFYGSTVGLAPVKSMCSPSSGAVNEDHSKNPLGIASTIAHEMGHNLGMSHDTSMVFGVIFLLSSSCRYVLPDRFSSC
SKMDLETFLQNYDVRCLLNSPNEDDLFGGPVCGNAIVE
>Q92035-D1
EFIVTTRLGRVQGLRLPMPDRSHVVAFLGIPFAEPPVGKKRFKPAEPKKPWNDVFEATDYSNACYQYVDTSYPGFPG
TEMWNPNKIMSEDCLYLNVWVPASPRPHNLTVMVWIYGGGFYSGSSSLDVYDGRYLAHSEKVVVVSMNYRVGAF
GFLALNGSSEAPGNVGLLDQRMALQWVQDNIQYFGGNPKQVTIFGESAGGASVGMHLLSPESRPKFTRAIMQSGVP
NSPWATVSFDEARRRAIKLAKFVSCPDDDDAELVDCLRNKQPQELIDHEFRVLPYSSLFRFSFVPVIDGVVLPDTPDA
MLSSGNFKDTQILLGVNQDEGSYFLIYGAPGFSKDNDSLISREDFLQGVRMSVPHANDIGLEAVILQYTDWLDEDNP
LKNREAMDDIVGDHNVICPLQHFARSYANSAQQTGTTFQGNLVSVYLYLFDHRASNLAWPEWMGVIHGYEIEFVF
GLPLEKRLNYTAEEEKLSRRIMRYWANFARTGNPNINIDGSVESRRRWPLFTATEQKHVGLNTDAMKVHKGLKTQF
CALWNRFLPRLLNITDNIDDAERQWKAEFHRWSSYMMHWKSQFDHYSKQERCTDL
>Q9IAB0-D1
KYPEKLLFQLLLNGNNHTVHLQKNRLLIGRNYTEIHYEADGSTVITSPKLEDHCYYHGRIDGIDDSSVSVGVCSGMR
GFVRAEEQMYLIEPLGNSTEGDHAFYKREHLRRKRSAYGDSGITVYDTEPRTEALFKRSSMVYVSKFTPHITGISQLMI
YISLQYRRFKSNVDTVRARMLEAINHVDKFYRRHNIRVLLVGLEVWNVEDQFLVSVNDNETLTRFIQWRQKRLSNIV
KHDNAQLVTGVDFLDKTVGLANKFAMCTKASGGINQDHSTSPLGLAATIAHEMGHNMGMSHDVRGCTCGLSDCI
MTEDMNSAASVFPELFSDCSLDQLQIFLENVNPICLLDRPSSDKIYGGPVCGNAFLDPGEECDCGTEEECNNPCCNA
KTCKFTEGSFCAQGECCENCQVEYTGRVCRASVNDCDLDEYCTGMSEKCPQDSF
>CL3069.Contig2-D1
WAWHLSVIFLSLILIAHHDVSANRCAASKATSCSACLQTGFGCAYCPDEVFGFDRCDLLENLKSHGCVQIVSVQSSM
SMLKNIEINKNLKHSQVAPQQMSMTLLPGEEREVEMEVFEPARGPLDLYILMDFSNSMSDDLDNLKKMGDELARLV
GTLSDDYTIGFGKFVDKVTEPQTDMRPSKLKEPWAKSDPPFSFQHVITLTSNISTFRQILQKERISGNLDAPEGGFDAIL
QAAVCQSQIGWRQHSTHLLVFSTESAFHYEGDGINVLAGILPRNDEACHLDTKGLYTHATQQDYPSVPTLVRLLVQ
NNIIPIFAITNHSLSYYEMLLHYFPIAELGVLTDDSSNILNIIKNAFDSIRSKISIQTENKPKAIISQILSASGVASDYGKFKI
QPGEIGKFKVLLSAKTSVNDKPVCSLNTNERTGTIRVKPTTFSSALEIQTTVLCETCNCEQFPITKASRCSGNGDLVCG
TCKCHNNWLGPFCNCSSQMSSDVSGCVAPGSTQPCNGRGDCLCGTCLCYNPNQYEGQFCQYDRSQCHRSEDDSY
RGRCYMGGCVCDSGWTGNACECPLSNATCLDNKGGLCNGHGVCKCGRCRVIRCDCTSKMWECLGVILCGGCEC
EYAGLELGTTCEPNFQAQLGMCESRRSCVQCQAWKTGEIKGDKCKECPFTIKMVDELKDRDKVIETCEYRDEEDDC
TYHYTVNYPNNPSDKEHEVEVLKKKDCPPGGFLWLIPLIMFLMLLLGLLLLCCWKYCACCKACLARLCVCSPCCAM
GRIVGFKEDHYMLHQSMLSSDHLDTPMVRTGPTNSTDVVRWKVKDNVHRGPNLPQNQIQPNPKEIIDVPLSLRLHR
AFSDELSRPEARNTEMLKQEVEENLNNVFRQIPGAHRVQKTRFRTQRNAGKRQNNTIVDTVLSAPRSSYHNITNLV
HKQVQSGNFSDLKVVPGYYTVATDREAMGVVELQEDVESVDVRVPLFIKDEDDDKKQLLVEAIDVPMGIANIGKRY
VNITVIKEHAKSILTFLQPSYTFSRQDGVANIPISRDIIEDGRTQFTYCTRDLTAKDKKDYISVDGDLMFQPGETQKMVP
VKLLELSEGDALLDKQPKQFVMDLSNPLQGAKLGKYPRTTINIADKPESSVIMFKNSTQNFNISDALYTVPVIRTQGL
ENPSTVNWRTISSRFNLSGPVKFAPGEMEKNIVIDTRTQQMVPKESFQLELFDPSANSLIRDRKTTLVNITDSRGDFVS
KTGSVPVKASSPTGRIFAPTNIEATPTGPKKIHLNWNPQPGAKGYKVKYWIYGDPEAEAQVVDVKNTHAELTNLYP
YCDYEMRVCSYNAQGDGDYSDITQCQTLEDVPSEPGRLAFNVIGQTVTQLSWAEPAEPNGVITEYEVVYTPINEDSK
PIGPDKKVKIDNPKKRMLLIENLQKSQTYRYKVRASNKVGWGPYRDATINLATQPLRPMSIPIIPDIPIVDAEAGDDY
DSFLMYSNEVLRSPTSSTRPSVSEFSEEQIINGKWDQGFLFPGGSGSMSRNISTSSSYNLSPRPGGANQTVETTMTYITN
KGGSMPRKHDIHTEDVTLRKRSENRNYYDNDGIRDSIVMSELTGGFSEVLSGSSFSQSTTTSYSMNSRVHNHSDDVNE
ALQNLDRVLQETRLQRGVPETPSRLVFSALGPTALKVSWQEPHCETPIRRYCVLYQLLSGGEIKSIDINNPTQNSVMV
QDLLPNQSYLFKVKAESHEGWGPEREGVITIESAVDPKSPLSPVPGSSFTLSTPSAPGPLVFTALSPETLQLSWDKPRKP
NGEIVGYVVTCEQLHGGGDQRSFQLSGNSATTLTVSDLSENVPYKFKVQAQTTQGFGPEREGIITIESQDGSVGQYGS
QSVMRREVFNMPSQSTTQTTHTMFTDPFITPEGIMMSGRQVTQHSEISGSITRQVEMVQRGVKSTVTKKQYYEA
>CL5628-D1
MFSFVDIRLGLLLAATVLVVRGQGEDDSIYNSCQLEGQSYNDKDVWKPEPCRICVCDSGTVMCDEVICEDITDCANP
EIPDGECCPICIDGTESPQISGPNGDKGDPGPKGDPGPVGQPGNDGIPGENGLPGPPGPPGPPGLGGYNLPQLSYGSE
KSSGGQAFPGPPGPMGPRGPPGSSGSPGPQGFTGPPGEPGEPGSPGPMGPRGSPGPPGKNGDDGEAGKSGRPGERG
AAGAQGARGFPGTPGLPGIKGHRGFNGLDGAKGDSGPAGPKGEPGSPGENGVAGVMGPRGLPGERGRPGAAGPS
GARGNDGNSGPAGSPGPTGPSGPPGFPGAAGAKGETGPAGGRGSEGPQGARGEPGNPGPAGAAGPPGPPGTDGS

AGNKGSPGAAGITGAPGFPGTRGPAGPAGPLGAVGPKGLNGDAGAPGNKGEPGPKGEPGPVGPQGLSGPPGDEG
KRGARGEPGGPGPLGPPGARGAPGNRGFPGAEGGPGPKATPGELGPNGPAGAQGATGESGRPGEPGLPGSILVLFLI
LMNMSTFLVLSYSPVSPIKGVTGSPGAPGPDGKAGPAGAAGQDGASGAPGPAGSRGLPGVMGFPGPKGADGEPGK
PGERGVEGPTGSVGAPGKDGDVGAPGPSGPAGPSGEKGEQGPAGGPGFQGLPGPQGSTGETGKAGDQGLPGEAGV
HGPPGPRGDRGLPGERGTPGAVGPTGSRGSPGSPGNDGARGDPGAAGAPGGVGGAGPQGMPGERGSSGLPGARG
DRGDGGPKGPDGAPGKDGVRGLTGAIGSPGPSGTSGEKGEPGPVGPPGSTGPRGGPGDRGEPGSPGPAGFAGPPGA
AGQPGAKGEHGDSGPKGDAGAPGPPGPVGPAGHQGPPGATGPKGARGGAGPPWTGFPGAAGRVGPPGPSGVAG
PPGPTGPGGKEGPRGARGEKGAPGRPGEVGGVGAPGPSGERGSAGADGPPGPPGAPGPQGINGGGGIAGGPGQRG
ERGMPGPSGPSGEPGKQGVTGPAGERGPPGPMGPPGLSGGPGEAGREGSVGHDGAPGRDGAPGPKGDRGESGHPG
APGAPGLPGPPGPVGSSGKPGDRGETGPAGPAGISGPAGARGALGPAGPRGDKGEAGETGERGMKGHRGFSGMSG
LPGPAGHPGEPGPAGPTGPAGPRGPAGSTGAHGKDGMNGIPGPVGPPGPRGRTGEMGAAGAPGLPGPPGPPGPA
GIGEPFPIMPQPEKAADPYHGYRADEASVQDRDAEVDSSLKTISQKIENILSPEGTKTNPARMCRDLRMCHPEWKSG
SYWVDPNQGSPLDAIKVFCNMETGETCVNPTRASIPLKNWFISKNIREKKHVWFGESMPDGFQFQYGSEGADSEDVS
IQMTFMRLMSNKASQNITYHCKNSIA
>CL5628-D2
MFSFVDIRLALLLSATVLLARGQGEDDRTGSSCTLDGQVYNDRDVWKPEPCQICVCDSGTVMCDEVICEDTSDCAN
PVIPPDECCPVCPDDGKSYFLPKASVSHTLSHLLFCFQGLPGPPGNDGIPGQPGLPGPPGPPGPPGLGGNFSPQLSGG
YDEKSGGAAMAVPGPMGPMGPRGPPGPPGLSGPQGFTGPPGEPGEPGATGAMGPRGPAGPPGKNGEDGESGKPG
RPGDRGPAGPQGARGFPGTPGLPGIKGHRGFSGLDGAKGDSGPAGPKGEAGAPGENGTPGAMGPRGLPGERGRA
GANGAAGARGNDGAAGAAGPPGPTGPAGPPGFPGGPGSKGEVGPQGARGGEGPQGARGEPGSPGPAGAAGPAG
NNGADGAPGAKGAPGAAGIAGAPGFPGPRGPPGPNGAPGAVGPKGNTGEGGAPGAKGDAGAKGETGAPGIQGP
PGAAGEEGKRGARGEPGPIGARGPPGERGAPGGRGFPGADGAAGPKGGPGERGGPGVVGPKGASGEPGRNGEPG
MPGSKGMTGSPGSPGPDGKMGPTGPAGQDGRPGPPGPVGGRGQPGVMGFPGPKGSAGEAGKPGERGVMGAIGAP
GASGKDGDVGAPGAPGPAGPAGERGEQGPSGSPGFQGLPGPQGATGEPGKPGEQGLPGEAGAPGVSGARGDRGFP
GERGAPGIAGPAGPRGSPGSAGNDGAKGEPGAPGAPGAQGAPGLQGMPGDRGAAGLPGLKGDRGDQGAKGTDG
APGKDGIRGLTGPIGPPGPAGAQGDKGEPGAAGPLGPTGARGPPGERGETGAPGPAGFAGPPGTDGQAGAKGESG
DTGAKGDAGAPGPAGATGAPGPQGPVGSTGAKGARGPAGPPGATGFPGAAGRLGPPGPAGNAGPPGPPGAPGKE
GAKGLRGETGPSGRTGEVGAPGAPGAPGEKGPPGAEGPAGSAGIPGPQGLAGQRGIVGFPGQKGERGFNGLPGPSG
EPGKQGPGGPSGERGPPGPMGPPGLAGPPGEPGREGTPGNEGSPGRDGAAGPKGDRGESGAAGAPGAPGPPGAPG
PIGPAGKTGDRGESGPAGPAGAAGPSGPRGPSGPAGARGDKGETGEAGERGMKGHRGFTGMQGPPGPPGPPGESG
PAGSSGPAGPRGPVGASGTSGKDGMSGLPGPIGPPGPRGRNGEIGPAGPPGPPGPPGPPGPSGGGFDIGFIAAPQEKA
PDPFRHYRADDANVMRDRDMEVDTTLKSLSQQIENIRSPDGTKKNPARTCRDLKMCHPDWKSGEYWIDPDQGCN
QDAIKVYCNMETGETCVYPSEADIPKKSWYTSKNIKEKKHVWFGEAMTDGFQFEYGSEGSNPEDVNIQLTFLRLMST
EASQNITYHCKNSIA
>CL5628-D3
MLRFVDSRTVLLLVATQFILQAVVRCQQEDDQEDLGGCIQDGQHFEDRAVWKPEACRVCVCDSGAVLCDEVICEEL
RDCNNPIIPPGECCPICPADQDQTSEQGPRGDRGPKGEKGIPGPRGRDGEPGTPGNPGPPGPPGPPGPNPPGLGGNF
AAQMAVGFDEKAGGATMGVMQGPMGPMGPRGPPGPSGSPGPQGFQGSPGEAGEPGPSGPMGPRGPPGSPGKPGS
DGENGKPGKPGDRGPTGSQGARGFPGTPGLPGIKGHRGHPGLDGAKGEIGAAGAKGETGASGENGAPGPMGPRG
LPGERGRPGPSGAAGARGNDGLPGPAGPPGPVGPSGSPGFPGSPGSKGEAGPTGHRGAEGAQGPRGEAGTPGSPGP
AGAGGNPGTDGIPGAKGSAGAPGIAGAPGFPGPRGPPGPQGATGPLGPKGQSGDPGIPGFKGEAGPKGERGIVGPQ
GAPGPIGEEGKRGPRGEPGSAGPLGPPGERGAPGNRGFPGQDGLAGAKGAPGDRGVSGLTGPKGGNGDPGRPGEP
GLPGARGLTGRPGDAGPQGKVGPSGAPGEDGRPGPPGPQGTRGQPGVMGFPGPKGASGEPGKPGEKGLIGSIGVRG
LPGKDGETGAAGPPGPAGPAGERGEQGQPGPPGFQGLPGPSGPPGEGGKPGDQGVPGEGGVPGVVGPRGERGFPG
ERGSAGAQGLQGPRGLPGTPGTDGPKGAIGPAGSAGAMGPPGLQGMPGERGTAGISGPKGDRGDTGEKGPEGAPG
KDGSRGLTGPIGPPGPSGPNGAKGETGAIGPSGPAGTRGAPGDRGEVGPPGPAGFAGPPGGDGQPGIKGEQGESGQ
KGDAGSPGPQGPSGAPGPVGPTGVSGPKGARGAQGAPGATGFPGAAGRVGPPGPNGNPGSAGPAGPAGKDGPKG
VRGDAGPPGRPGDVGLRGAPGSPGEKGEPGPEGQHGPLGPPGPSGLAGQRGIVGLPGQRGERGFPGLPGPSGEPGK
QGSPGSGGERGPPGPIGPPGITGPAGELGREGNPGSDGPPGRDGAPGVKGERGNTGPIGAPGAPGAPGSPGPVGPVG
KQGDRGENGPQGPAGAPGTAGARGMAGPQGPRGDKGESGEVGERGQKGHRGFTGLQGLPGPPGSPGDQGASGP
SGPSGPKGPPGPVGPAGKDGANGIPGPIGPPGPRGRSGESGTAGPPGNPGPPGPPGPPGPGIDMSAFAGLGHTEKGP

DPMRYMRADEASNNLRQHDVEVDATLKSINSQIEDIRSPDGSRKNPARSCHDLKICHPDWKSGEYWVDPNLGCTA
DAIKVFCNMETGETCVKPTTSNIPRKNWWSIKSKSQKHVWFGENMGGGFHFSYGEKIPTPNVASIQINFLRLLSSEAS
QSITYHCKNSVA
>CL7398-D1
MWVSIGLIVALLFIALFLKYVFGNSGPSPFDVDMREPLKPVQLDKKERNKVLKQGFLASRVPQDLDAIVIGSGIGGLSI
AVLLAKVGKKVLVLEQHDRAGGCCHSFSEKGFEFDVGIHYIGNLEETGRFRCIVEQLTNGQLQWEPLENPFDQVVL
GPPENRRVYPIYSGNTRFPEELKKCFPGEEKAIDEFMRLVKNCGRGVWMVLLLKLLPSPVAKFLAYTGLANRMSYFFS
YGSRSLSEVVNSLTENKELRAVLCYIFGTYGNAPKEASFSMHSLLVCHYLPGAWYPKGGASQIAYNMIPIIEKAGGAV
LVRAPVSRILLNGANEAIGVSVMKGQEEVHVRAPIVISDAGIFNTYQQLLPKEVQTHAAIQKQLSMLKHGEGGLSIFI
GLNGSKEELGLKAENYWIFCENNLDELFNCYIKGEREDSTKNIPMIFVASPSAKDPTWEQRQPDKSTLSVVSFAPYSW
FEEWKDGKVKNRGDDYEELKETIINSVLELLTQIYPEIKDKIEYIEAGTPITNQHYLAAPKGEIYGADHSTSRFTAEVCV
TLRPQTPIKNLFLTGQDIFSCGFAGAIAGAMVCGSAILNRNLYRDIESLRKKLKNTNSKKVQ
>Unigene1108-D1
MDQRVASILLFLVALVAGHGERYMVKKVMKAPYAVKGHVVSVAGEPGAPGEPGEPGPPGPPGHPGENAVGLPGP
QGPPGPPGPSGYSAPGKPGTPGGPGKPGANGAPGPKGDTGSPGPQGPRGAPGPSGIPGPAGLSSPGKPGAAGMPGS
MGPRGETGPKGHPGIPGAPGQKGDRGIGIQGPPGETGSTGPMGPVGAPGQPGVGKPGKPGYPGEPGKPGSSGLDGT
PGPMGPTGPKGHTGAPGIGMPGKPGENGAPGLPGPSGLKGPQGATGAPGAPGSPGYGKPGAPGFKGDTGPVGSPG
ATGQKGEAGARGATGYTGATGPMGPAGSQGPRGFPGEKGVTGEKGETGPMGPQGFKGHKGDQGPQGPEGKSGY
PGAAGPQGPRGATGAPGSKGETGEAGATGAPGAPGPVGPKGHTGNSGPAGETGPAGAPGSRGPSGPSGPPGAPGA
KGHPGLPGSPGPAGLAAKGIPGPQGPPGLPGSDGAPGETGPAGPPGPPGPPGEVIIAETKGTFVNEPFVKTPMSAFSA
LTTTPYPPAGSPVKFEQVVYNAENHYDPESGIFTCQVPGVYFFSYSMHVNGANALVALYKNEEPIMFTYDEYNKGFL
DQMSGSTVLQLNEQDTVYIQIPDDEANGVFAADNVHCSFSGFLIAST
>Unigene1108-D2
MELHTVCVLLLLAVCVRATPDRYYIKKQQSFPVKGQELSGAPGIPGEPGPPGPPGPPGPPGMSIMGRQGQPGPPGPA
GPAGYAAPGKHGSPGAPGKPGDNGMPGERGDSGPVGPQGARGPPGAPGSPGPAGFSAPGKPGPHGLPGAMGPRG
EPGSKGLSGIPGLPGQKGEPGHGVQGHPGGPGPIGPSGPPGQPGQPGIGKPGATGYTGEPGKPGLPGRVGAPGPMG
APGPKGHQGAPGIGAPGNSGQNGAPGLPGPMGPKGFQGPAGQPGAPGLQGVGKTGAPGIPGNRGAPGTPGTSGQ
KGEPGTTGFTGQPGASGPVGPTGPQGDRGFPGETGPLGPKGDAGIIGAPGSKGQKGDLGPQGFTGKPGIPGASGPQ
GPHGATGYPGPKGDRGYTGPSGSPGAMGPTGLKGHTGPQGMPGNRGENGLPGARGPMGPAGTSGSPGAKGNPG
LPGPPGPAGLVTKGLSGPQGPPGFPGARGQDGRPGLAGPPGPPGPPGETVYYNEKSMPLKSEVYPVSHDLMKPRMS
AFTALLTTAYPNSGTPIVFNQIVYNGENHYDPSTGVFTCQVPGFYYFVFHMHVNGANALVALYKNNEPVVFSYDEY
NKGFLDQMSASTVLMLHMGDTVYVQVPDDQSNGIYADNNVHCSFTGFLIAST
>Un37646-D2
MRSILAFMVLGACTLSAWSKPINSRHVSFPGDILKNMTDIQMADSYLERFGYKRILDKSGRQGAVLTHKALRRLQTQ
LGLEETGELDQPTIDAMKKPRCGVPDVRNYQTFDGDLKWDHNDVTYRILNYSPDLDVSTIDDAFARAFKVWSDVTP
LTFRRLYDGTADIMISFGKKDHGDPYPFDGKDGLLAHAYPPGEGVQGDAHFDDDEYWTLGKGPAIKTYFGNADGA
LCNFPFRFEGKSYSSCTTEGREDGLPWCATTANFDKDKKYGFCPSELLYTFDGNADGVPCVFPFVFEGKTYTSCTTEG
RDDGYRWCATTSSFDKDKKYGFCPNRDTAVIGGNSEGEPCQFPFIFLEKSFTSCTSEGRSDGKLWCATTSNYDKDQK
WGFCPDKGYSLFLVAAHEFGHALGLDHSNIQDALMYPMYKYIADFSLHQDDIEGIQYLYGPKKGPKPTPPKPSTTTA
STISTAKPTKKTPKTTPSTASTTTPSVFTPVDPSVDPCTADRFDAITEIQGELHFFKDGYYWTSSSSGNKERKGPFLVSER
WPGLPAKLDTAFEDPITKQMYFFAETQFWEFTGKNVRGPRSIEKLGLPANVDKVEGSLQRGKGKVLLFSGENYWRM
DLKTQQMDKGFPQQTDMTFGGVPVDAHDVFLFKGNYYFCRNIYYWRMTSKRQVDRVGYVYELLNCPDY
>Un37646-D3
LKKMQKFFALPETGEIDQNTVEIMKKPRCGVPDVANYNFFPSKPKWQNNEITYRILGHSPDMDEETIDDAFFRAFKV
WSDVTPLKFTRIMSGDADIMINFGRNEHGDGYPFDGKDGLLAHAFAPGPGIGGDSHFDDDEHWTLGDGQVVKVKF
GNAEGEFCKFPFLFMGKLYNSCTNQGRDDGFLWCSTTYDFDKDGKYGFCPHELLLTLGGNGDGAPCKFPFTFQGEK
YDSCTTSGRDDGYRWCATTENYDQDKTYGFCPETALSTIGGNSEGAPCVFPFIFLGKSYDACTTSGRNDGKMWCSST
KSFDEDRKWGFCPDQGYSLFLVAAHEFGHALGLEHSQDPGALMAPIYTYTKHFRLSNDDIRGIQELYGAPTGKPLPP
TQGPVTPMDICSENIIFDAIAQFRGETFFFTDRFLFRTTNAEKKPSGPLLVATFWSELPEKIDAAYENPLEEKSVFFSGDE
MWVYSASTLERGYPKKISSLGLPSDLQHIDAAYAFHKSKKTYLFAGDKFWRYNEAKNKMDPGFPKLIADSWNGVP
DDLDAAFSTSGQGYSYFFKGSHYHKMDENLRIVKVGEV

>Unigene40283-D1
MLSFVDTRILLLLAVTSYLASCQWPRGDKGPRGDRGPKGPDGKPGKHGLPGPPGPPGPPGLSGNFAAQYDGSKGPD
PGPGPIGLMGPRGPSGPPGTPGPQGPQGHAGEPGEPGQAGPGGPRGPPGPPGKSGEDGNNGRPGQPGDRGTPGSQ
GARGFPGTPGLPGMKGHRGYNGLDGRKGEPGEMGTKGETGAHGSNGTPGQRGARGLPGERGRPGPPGPAGARG
ADGNTGPSGPAGPLGAAGPPGFPGGPGPKGEMGPAGPSGPSGPQGQRGEPGTNGVSGPVGPPGNPGANGLNGAK
GAAGTPGVAGTPGFPGPRGGPGPQGPAGPSGPRGLSGDPGPVGVKGESGAKGEPGNIGAQGPTGAAGDEGKRGST
GEQGSAGPVGLRGARGAAGTRGLPGLAGRGGSMGMPGARGAAGAPGARGPPGDAGRAGEAGLVGARGLPGSPG
SPGPQGKEGPAGPSGQDGRSGPPGPTGPRGQPGNIGFPGPKGPSGEPGKPGEKGLAGAPGLRGPPGSDGNNGPAGP
VGLAGGPGEKGEAGPAGAPGFQGLPGPAGPVGETGKPGDRGIPGDQGVAGPAGGKGERGNPGPAGASGAQGPTG
PRGPSGTPGPDGNKGEPGSAGLAGAQGPQGSVGMPGERGGAGTPGVKGEKGEPGYRGPEGNAGRDGSRGAPGPI
GPP
